# Glutathione-functionalized PVC for hydrogen-bonding assisted fluorescein adsorption and radical scavenging

**DOI:** 10.1039/d6ra07765j

**Published:** 2026-10-30

**Authors:** Alessio Giove, Amar Raj, Nihal Chelampetty Para, Matti Haukka

**Affiliations:** a University of Jyväskylä, Department of Chemistry 40014 Jyväskylä Finland alessio.a.giove@jyu.fi matti.o.haukka@jyu.fi; b University of Jyväskylä Nanoscience Center 40014 Jyväskylä Finland

## Abstract

The protection of aquatic ecosystems from the rising discharge of hazardous industrial dyes is a critical challenge for global environmental sustainability. In this context, a glutathione-functionalized polyvinyl chloride (PVC-GSH) adsorbent was synthesized *via* a nucleophilic substitution reaction and primarily evaluated for the removal of fluorescein. The integration of the GSH moieties into PVC backbone was confirmed by FTIR and ^13^C CPMAS NMR spectroscopies, complemented by morphological and thermal characterizations (SEM, BET, DSC, and TGA). The resulting PVC-GSH exhibited high selectivity for fluorescein and a rapid optical response, as evidenced by UV-vis and TRFE spectroscopies. Notably, PVC-GSH achieved a fluorescein uptake of ∼97 mg g^−1^ (highest experimentally observed) at pH 6.0, with equilibrium data best described by an empirical Freundlich model. TRFE measurements demonstrated a rapid signal response on a timescale of ∼3 s. Post-adsorption analysis through FTIR and XPS indicated that binding likely occurs *via* hydrogen bonding mediated by the GSH moieties, with possible contributions from secondary non-covalent interactions. Beyond its dye remediation capabilities, this novel material showed radical-scavenging activity, yielding a DPPH radical scavenging EC_50_ of 774 mg L^−1^. These results highlight PVC-GSH as a dual-function material combining rapid fluorescein uptake with antioxidant properties, and as a potential route for PVC upcycling.

## Abstract 2

Bio-functionalization of PVC with glutathione *via* nucleophilic substitution enables rapid, hydrogen-bonding-assisted fluorescein adsorption.

## Graphical abstract

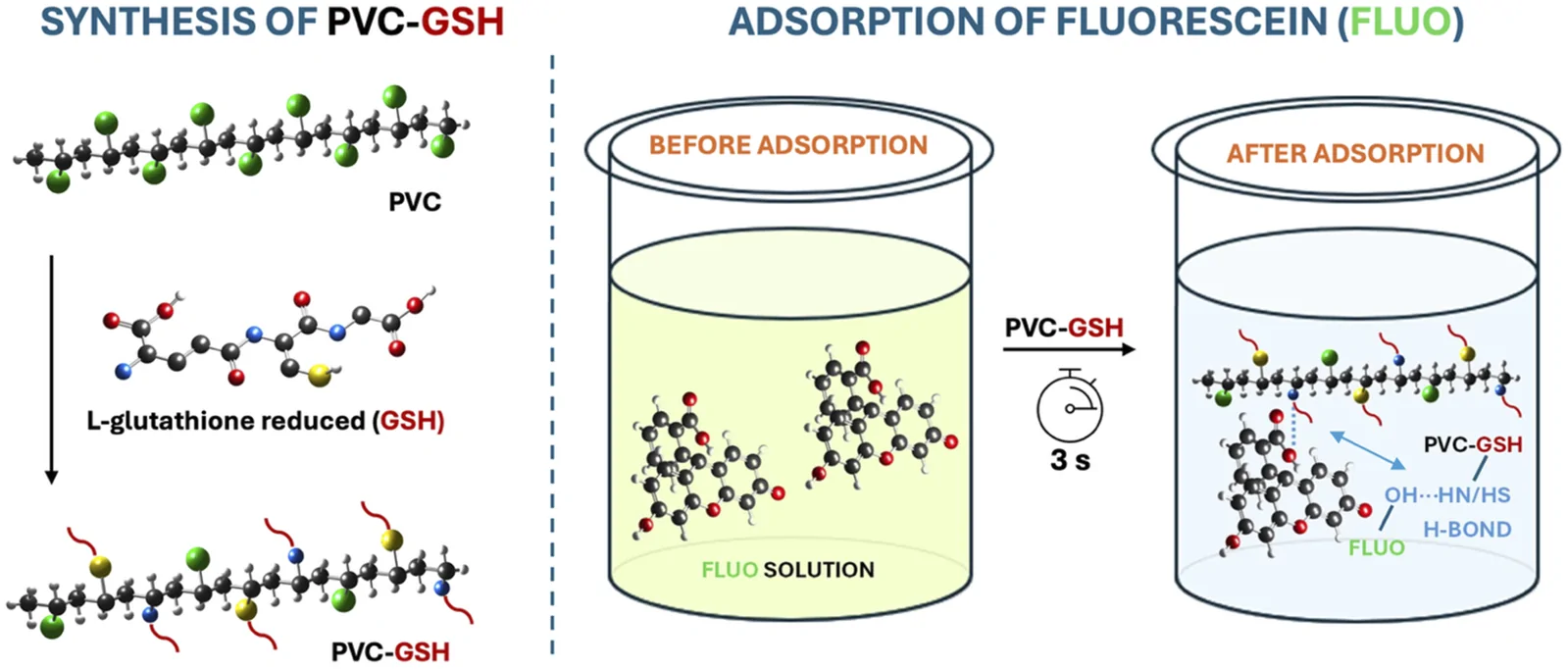

## 1 Introduction

The rapid expansion of textile, tanning, and printing sectors has resulted in the environmental challenge of organic dye contamination in wastewater effluents.^1^ The majority of organic dyes with industrial applications fall in the categories of azo^2,3^ and anthraquinone^4,5^ compounds. These are environmentally persistent and often linked to severe health risks, including carcinogenicity and mutagenicity, thereby posing significant threats to both human health and aquatic ecosystems.^6,7^ To address this issue, a range of traditional methodologies, such as membrane processes,^8^ electrochemical technologies,^9^ and advanced oxidation processes,^10^ have been employed. However, these conventional approaches are frequently limited by significant operational and environmental drawbacks. These include high operational and maintenance costs, significant energy consumption, reliance on toxic organic solvents, and the generation of secondary sludge that requires further expensive disposal.^11–14^ Consequently, there is urgency to develop more cost-effective, environmentally friendly, and highly efficient alternatives for dyes remediation.

Polymer-based adsorbents have emerged as compelling and sustainable alternatives. Polymeric materials offer diverse advantages, including low cost, scalability, and, in some cases, a tunable surface chemistry that enables easy functionalization.^15–17^ New materials with high selectivity and enhanced adsorption capacity toward targeted contaminants can be designed by covalently anchoring specific functional groups onto the polymer backbone.^18–21^ In this context, poly(vinyl chloride) (PVC) has been selected as the polymeric substrate to be modified. Owing to its cost-effectiveness and to its thermal, mechanical and chemical stability,^22,23^ PVC stands as one of the most widely applied thermoplastic polymers. When designing the modification of PVC, the focus is set to its labile Cl atoms, which can act as leaving group in substitution reaction.^24^ This inherent reactivity of PVC makes nucleophilic substitution a highly effective strategy for the chemical modification and post-functionalization of its structure.^25^ Furthermore, given that PVC is non-biodegradable and generates a significant waste stream, its chemical transformation into high-value functional adsorbents presents a direct pathway for upcycling waste PVC, simultaneously mitigating waste burden and creating advanced materials.^26,27^

In this work, we applied l-glutathione reduced (GSH), a naturally occurring, biobased tripeptide for the functionalization of pristine PVC. GSH is indispensable in biological systems, known for its roles as a powerful antioxidant^28,29^ and as an effective chelating agent.^30–32^ The thiol and amino groups of the cysteine and glutamate residues make GSH a strong nucleophile.^33^ Therefore, GSH is expected to be a highly effective molecule for the modification of PVC *via* nucleophilic substitution of its Cl atoms. To the best of our knowledge and based on a comprehensive literature survey, this functionalization represents a novel synthesis, as no direct reports regarding the modification of PVC with GSH *via* nucleophilic substitution have been documented to date. In detail, this study focuses on the synthesis and characterization of a novel PVC-GSH material. The dye adsorption capacity of PVC-GSH was initially evaluated by screening a selection of industrially relevant dyes, followed by a comprehensive series of batch adsorption experiments on fluorescein. The interaction between PVC-GSH and fluorescein was then investigated by combining adsorption isotherms with FTIR and XPS analyses. Beyond its primary application in water remediation, the antioxidant potential of PVC-GSH was assessed using the DPPH radical scavenging assay as a model test.

## 2 Experimental

### 2.1 Materials

The following reagents were used as received from the supplier, without further purification. l-Glutathione reduced (GSH, >98%) was purchased from Thermo Fisher Scientific. Acetone (≥99.8%), ethanol (technical grade), and chloroform (≥99.8%) were purchased from VWR. Poly(vinyl chloride) (PVC, low molecular weight), dimethyl sulfoxide (DMSO, for synthesis), tetrabutylammonium hydroxide (TBAOH, 1.0 M in methanol), fluorescein (free acid), crystal violet (≥90%), rhodamine B (≥95%), methylene blue (≥82%), rose bengal (95%), methyl orange (85%), and 2,2-diphenyl-1-picrylhydrazyl (DPPH) were supplied by Sigma-Aldrich/Merck. Deionized water was used in all adsorption experiments.

### 2.2 Equipment and characterization methods

Fourier transform infrared (FTIR) spectra were collected using a Bruker Alpha device equipped with an attenuated total reflectance module. Each spectrum was the average of 24 scans in the 4000–400 cm^−1^ range with a resolution of 4 cm^−1^.

^13^C CPMAS NMR spectra were recorded at 25 °C using a Bruker Avance 400 MHz spectrometer equipped with a CPMAS probe. The spinning speed was 8.0 kHz, with a CP contact time of 2 ms and a relaxation delay of 5 s.

UV-vis absorption spectra were recorded on a PerkinElmer Lambda 650 spectrophotometer using a quartz cuvette with a 2.5 mm optical path length. Measurements were performed at room temperature under identical conditions for all samples unless differently mentioned.

Time resolved fluorescence emission (TRFE) spectra were collected using a Horiba Aqualog spectrometer with a quartz cuvette of 10 mm path length, with stirrer adapter attached. For adsorption kinetics experiments, the initial fluorescence baseline was recorded for 20 s, after which PVC-GSH was introduced into the acquisition sequence. This approach allowed determination of the zero-time fluorescence signal corresponding to adsorbate addition. Spectral acquisition continued throughout the adsorption process with time resolution of 10 ms to monitor real-time changes in fluorescence intensity and spectral profile.

X-ray photoelectron spectroscopy (XPS) data were acquired using a Kratos AXIS Ultra DLD instrument equipped with a monochromated Al_Kα_ X-ray source (1486.7 eV) at 100 W. The spectral collection involved two main settings: survey spectra were obtained with a pass energy of 80 eV and a 1.0 eV step size, whereas high-resolution spectra employed a 20 eV pass energy and a 0.1 eV step size. To ensure both surface homogeneity and accurate charge compensation, both sets of data were measured at three separate spots on each sample. High resolution spectra were charge-corrected with respect to the position of C–C bonding in the C 1s high resolution spectra. The elemental ratios presented here were calculated as average of three positions of the sample. Standard deviation was determined according to the values related to these three positions.

Scanning electron microscope (SEM) images were collected on a Zeiss EVO-50XVP microscope. Samples for SEM analysis were coated with gold to increase their conductivity.

Nitrogen adsorption–desorption experiments were conducted using an Anton Paar Autosorb 6X00 analyser. Before being analysed, samples were degassed under vacuum at 110 °C. BET^34^ and BJH^35^ models were applied to determine the specific surface area and pore size distribution.

Thermogravimetric analyses (TGA) were carried out on a PerkinElmer STA 600 instrument. Prior to analysis, all samples were dried, and 7–9 mg aliquots were used for each measurement. The samples mass loss was recorded across the 20–600 °C temperature range within an open platinum crucible under an air flow rate of 40 mL min^−1^.

The thermal transitions of polymers were investigated using a PerkinElmer 8500 series power-compensation differential scanning calorimeter (DSC). All measurements were conducted under a nitrogen atmosphere with a flow rate of 50 mL min^−1^ to maintain an inert environment. For each analysis, approximately 3–4 mg of the material was encapsulated in 50 µL aluminium pans sealed with 30 µL pinhole lids, providing an effective volume of 20 µL. The instrument underwent temperature calibration using *n*-decane and indium standards, while energy calibration was performed using an indium standard. The thermal program involved heating and cooling scans at a constant rate of 10 °C min^−1^, from −20 °C to 150 °C. The glass transition temperature (*T*_g_) was determined at half heat capacity (*C*_p_) change.

Zeta potential (*ζ*) values of PVC-GSH and of the dye solutions were determined using a Zetasizer Ultra ZS90 instrument (Malvern Instruments Ltd, UK) operating in phase analysis light scattering mode. Measurements were performed at room temperature in deionized water (pH ∼7) following solution equilibration at 25 °C for 120 s. The electrophoretic mobility (*µ*) was converted to *ζ* using the Smoluchowski equation (eqn (1)).1
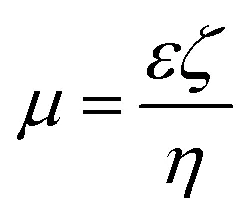
where *ε* and *η* represent the dielectric constant and absolute viscosity of the medium, respectively. Each reported *ζ* value represents the mean of three independent measurements, with 15 data accumulations per measurement, with error bars within ± 12%.

### 2.3 Synthesis of PVC-GSH

PVC-GSH was prepared with the following procedure. 4.0 g of PVC were dissolved in 40 mL of DMSO at 60 °C and kept under N_2_ atmosphere. In a separate flask, 0.4 g (1.30 mmol) of GSH were dissolved in 20 mL of DMSO and the solution was purged with N_2_ for 30 min. 2.60 mL (2.60 mmol) of TBAOH 1.0 M in methanol were then added to the GSH solution and the resulting mixture was stirred for 30 min at room temperature under N_2_ atmosphere. The activated GSH solution was added dropwise to the PVC solution and vigorously stirred at 60 °C under N_2_ for 18 h. After completion, the reaction mixture was cooled to room temperature and diluted with 60 mL of cold acetone to reduce viscosity. The polymer was precipitated by slowly pouring the diluted reaction mixture into a large excess (900 mL) of cold ethanol under vigorous stirring. The precipitated solid was collected by vacuum filtration and washed repeatedly with EtOH and with deionized water. The obtained material was air-dried and then transferred to a Soxhlet extractor. The Soxhlet extraction was carried out with a 1 : 1 v/v acetone:chloroform solution for 24 h. The purified material was dried under vacuum at 40 °C to constant weight, resulting in 3.2 g of dry PVC-GSH particles (73% w/w yield). PVC-GSH was finally ground using a mortar and pestle and sieved through a 200 µm stainless-steel sieve to obtain a uniform powder suitable for subsequent analyses and applications.

### 2.4 Adsorption properties of PVC-GSH

#### 2.4.1 Screening of dyes

The adsorption efficiency of PVC-GSH was evaluated against a series of dyes, including fluorescein, crystal violet, rhodamine B, methylene blue, rose bengal and methyl orange. Each sample was prepared in an Eppendorf tube by adding 0.2 mL of a 500 mg per L PVC-GSH stock solution (in DMSO) to 1.8 mL of dye solution (10% v/v DMSO/water). This resulted in a final concentration of 50 mg L^−1^ for PVC-GSH and 5 mg L^−1^ for the dye. The mixtures were shaken for 24 h and then centrifuged to separate the adsorbent from the liquid phase. The resulting supernatant was analyzed *via* UV-vis spectroscopy to measure the absorbance at the maximum wavelength of each dye before and after the adsorption process. The percentage of dye reduction was calculated according to eqn (2):2
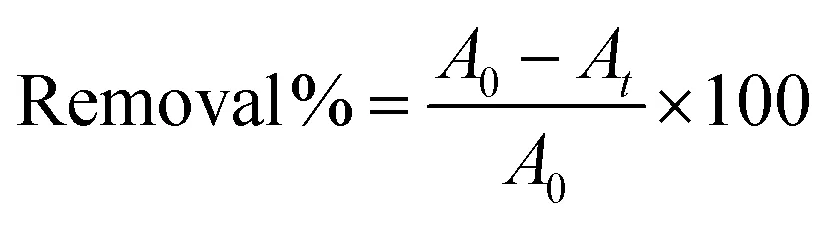
where *A*_0_ is the initial absorbance and *A*_*t*_ is the absorbance after the adsorption process.

Control experiments were performed under the same experimental conditions described above for the screening of dyes with PVC-GSH, using PVC as adsorbent at pH 5, 7 and 9. The pH value in control experiments was adjusted by using either 2 M HCl or 2 M NaOH.

#### 2.4.2 Adsorption of fluorescein

The adsorption of fluorescein with PVC-GSH in the different studies described below was performed as follows, unless otherwise stated. A stock dispersion of PVC-GSH was prepared in DMSO at a concentration of 2 g L^−1^ and stirred until a stable, homogeneous phase was obtained. For each experiment, 0.2 mL of this stock was added to a 10% v/v DMSO/water solution of fluorescein. The concentration of fluorescein and contact time were varied according to the specific study (effect of pH, kinetics or isotherms). After the adsorption process, the solid phase was separated by centrifugation, and the residual dye concentration in the supernatant was quantified *via* UV-vis spectroscopy. The concentration of fluorescein was determined through a calibration curve (*R*^2^ = 0.993, Fig. S1), prepared using standard solutions in the same matrix applied to the adsorption experiments. The equilibrium adsorption capacity (*q*_e_) was determined according to eqn (3):3
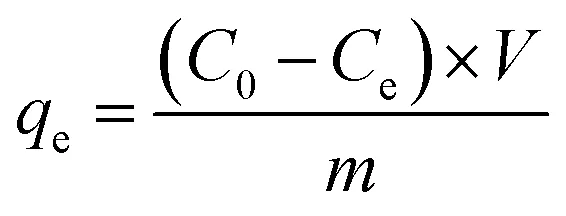
where *C*_0_ and *C*_e_ are the initial and equilibrium concentrations of fluorescein, *V* is the total volume of the solution and *m* is the mass of PVC-GSH.

#### 2.4.3 Effect of pH

The influence of pH on the adsorption efficiency of PVC-GSH was evaluated by performing adsorption experiments on 20 mg per L fluorescein solutions within the pH range from 2 to 12 with a contact time of 24 h. The pH value was adjusted by using either 2 M HCl or 2 M NaOH. The fluorescein removal percentage was determined for each pH value with eqn (2) by measuring the absorbance at the specific maximum wavelength corresponding to the dominant chemical species at each pH.

#### 2.4.4 Adsorption kinetics – UV-vis spectroscopy

The adsorption kinetics of PVC-GSH was initially studied by performing adsorption experiments on 6 mg L^−1^ and 45 mg per L fluorescein solutions with contact time ranging from 30 s to 11 min. The adsorption data were fitted using the pseudo-first-order^36^ (PFO, eqn (4)), pseudo-second-order^37^ (PSO, eqn (5)), Elovich^38^ (eqn (6)), intra-particle diffusion^39^ (IPD, eqn (7)) and Avrami^40^ (eqn (8)) kinetic models.

4 ln(*q*_e_ −*q*_*t*_) = ln(*q*_e_) − *k*_1_*t*

5
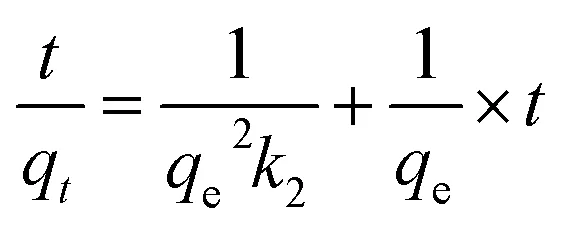
6
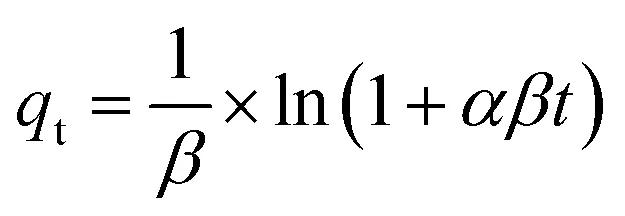

7 *q*_*t*_ = *k*_id_*t*^0.5^ + *C*

8 *q*_*t*_ = *q*_e_(1 − exp[−(*k*_av_*t*)^*n*^])

where *q*_*t*_ (mg g^−1^) is the adsorption capacity at time *t* (min), *k*_1_ (min^−1^) is the PFO rate constant, *k*_2_ (g mg^−1^ min^−1^) is the PSO rate constant, *α* (mg g^−1^ min^−1^) is the initial adsorption rate, *β* (g mg^−1^) is the desorption constant, *k*_id_ (mg g^−1^ min^−0.5^) is the IPD rate constant, *C* (mg g^−1^) is the boundary layer parameter, *k*_av_ is the Avrami kinetic constant and *n* is the Avrami exponent.

#### 2.4.5 Adsorption kinetics – fluorescence emission spectroscopy

Due to the high adsorption rate of PVC-GSH, the kinetic study was extended by using fluorescence emission spectroscopy to analyze the phenomena occurring during the first 30 s of contact time. In these experiments, the adsorption of fluorescein solutions with concentration ranging from 6 to 45 mg L^−1^ by PVC-GSH was monitored with 10 ms intervals, up to a maximum of 150 s of contact time.

#### 2.4.6 Adsorption isotherms

The adsorption isotherms were obtained by performing adsorption experiments with PVC-GSH (50 mg L^−1^) on fluorescein solutions with concentration up to 15 mg L^−1^. The experiments were conducted at pH 6, room temperature and with a contact time of 6 hours. To describe the adsorption process the experimental data were fitted using four isotherm models^41^ including Langmuir (eqn (9)), Freundlich (eqn (10)), Temkin (eqn (11)), and Dubinin–Radushkevich (D–R, eqn (12)).9
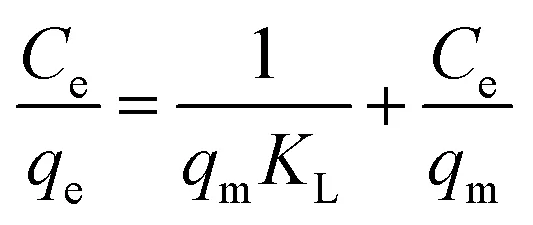
10
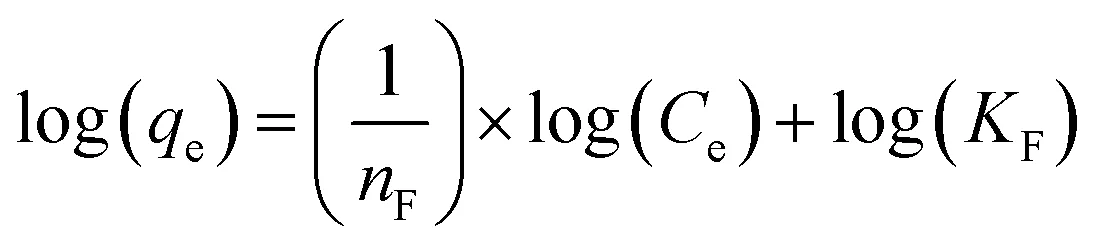
11
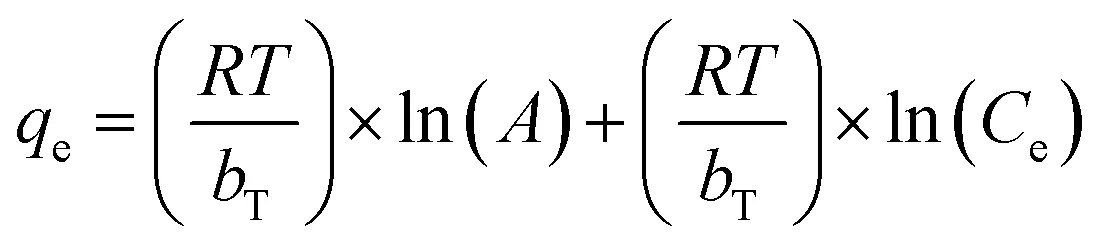

12 *q*_e_ = *q*_m_ × (−*K*_DR_ × *ε*^2^)

where *K*_L_ is the Langmuir adsorption constant, *K*_F_ is the Freundlich adsorption capacity, *n*_F_ is the Freundlich intensity parameter, *b*_T_ is the Temkin isotherm constant, *K*_DR_ is the D–R isotherm constant and *ε* is the Polanyi potential.

#### 2.4.7 Reusability and stability

The reusability of PVC-GSH was evaluated over four adsorption–desorption cycles. Adsorption experiments with PVC-GSH (1 g L^−1^) were performed on 2 mL of 25 mg per L fluorescein solutions at pH 6, room temperature and with a contact time of 30 min. In each cycle, following the adsorption process, PVC-GSH particles were separated by centrifugation, and the supernatant was analysed by UV-vis spectroscopy. The adsorbed fluorescein was then desorbed with 0.2 mL of 10^−2^ M NaOH for 10 min, and the adsorbent was washed three times with deionized water and dried before the next cycle. The removal efficiency of each cycle was normalised to that of the first cycle. The stability of PVC-GSH was assessed by stirring at 200 rpm 0.1 g of polymer particles in 10 mL of a 10% v/v DMSO/water solution for 24 h at room temperature. The solid was then recovered by centrifugation, rinsed with water, dried and analysed by FTIR.

### 2.5 Antioxidant properties of PVC-GSH

The antioxidant activity of PVC-GSH and PVC was evaluated using the DPPH free radical scavenging assay as follows. For the test of each material, two solutions were prepared: a 100 µM stock solution of DPPH in ethanol and a dispersion containing 2 g L^−1^ of polymer (either PVC or PVC-GSH) in DMSO. 100 µL of the DPPH solution were mixed with varying volumes of polymer solution and the total volume was kept constant at 1 mL with pure DMSO. This procedure yielded target concentrations of 200, 600, 1000, 1400, and 1800 mg L^−1^ of polymer. The resulting mixtures were incubated in the dark at room temperature for 15 minutes. Following incubation, the absorbance of each sample was measured at 524 nm using a UV-vis spectrophotometer. A control solution was prepared using 100 µL of DPPH solution and 900 µL of pure DMSO. The radical scavenging activity at each concentration of polymer was quantified as inhibition ratio using eqn (13):13
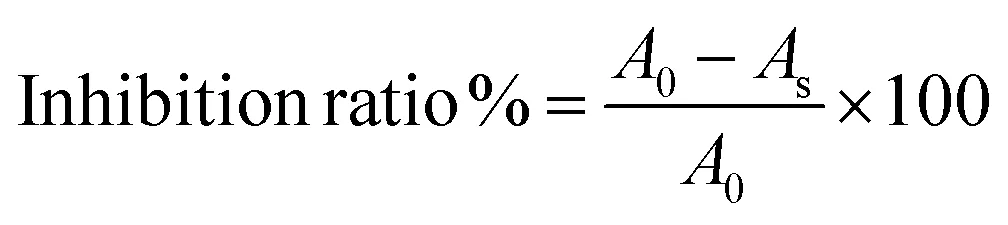
where *A*_0_ and *A*_s_ are the absorbance of the control solution and DPPH with polymer mixture, respectively.

## 3 Results and discussion

### 3.1 Synthesis and characterization of PVC-GSH

The chemical modification of PVC was performed by nucleophilic substitution of its Cl atoms with GSH moieties, which were connected through thiol and amino groups (Fig. 1a).

**Fig. 1:**
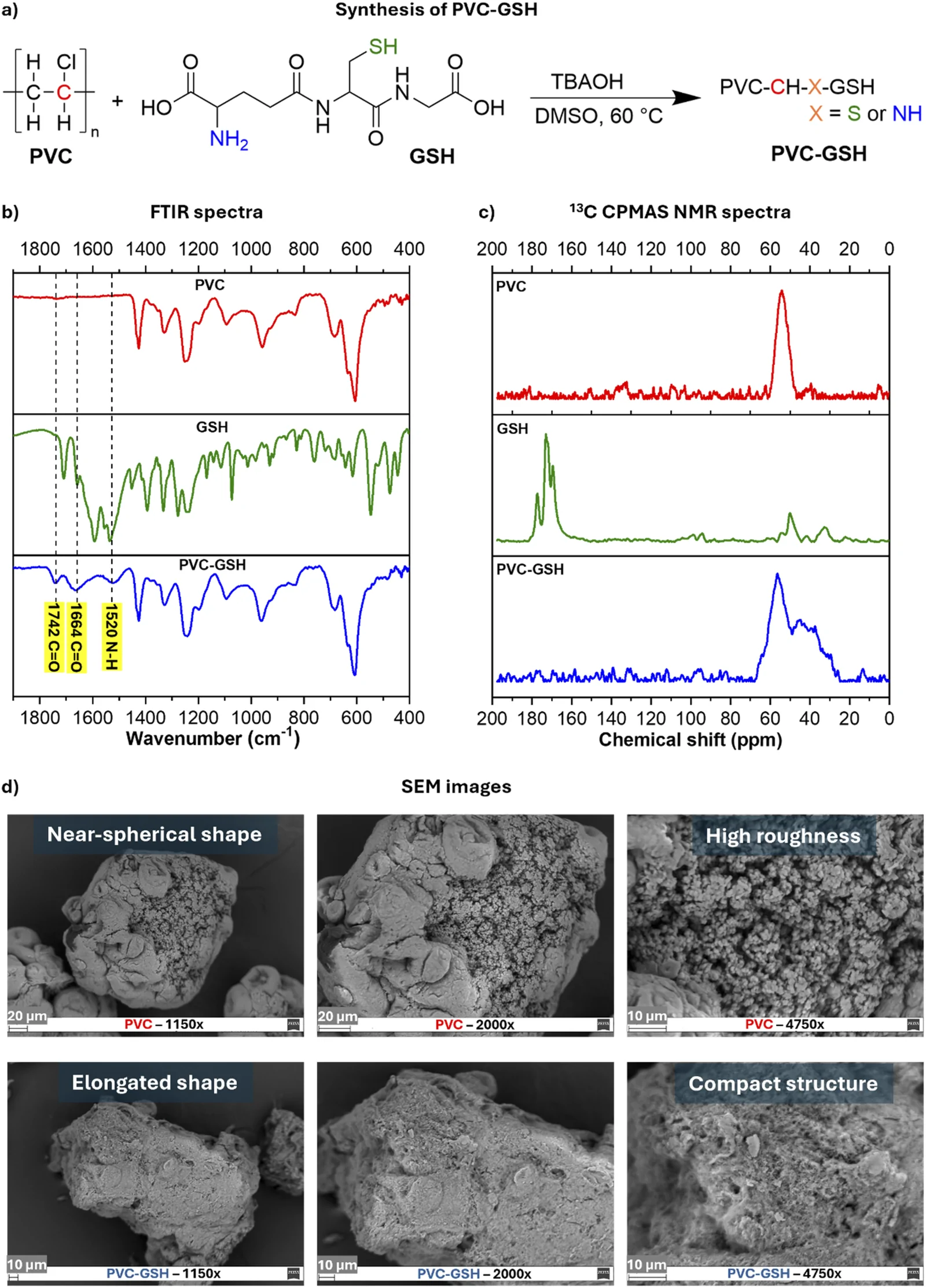
(a) Synthesis of PVC-GSH. (b) FTIR spectra of PVC, GSH and PVC-GSH. (c) ^13^C CPMAS NMR spectra of PVC, GSH and PVC-GSH. (d) SEM images of PVC and PVC-GSH.

The successful introduction of GSH onto the PVC structure was confirmed with FTIR (Fig. 1b) and ^13^C CPMAS NMR spectroscopies (Fig. 1c). The FTIR spectrum of PVC-GSH was compared with those of pure PVC and GSH, whose characteristic peak assignments are well-established in the literature.^42–44^ The FTIR spectrum of the PVC-GSH exhibited three characteristic bands of GSH moiety in the carbonyl/amide region at 1742 cm^−1^, 1664 cm^−1^, and 1520 cm^−1^. Specifically, the band observed at 1742 cm^−1^ is assigned to the C=O stretching vibration of the carboxylic acid groups of the glutamate and glycine residues, supporting the preservation of these terminal functionalities. The strong band at 1664 cm^−1^ is characteristic of the C=O stretching vibration within the peptide bonds (amide I) of the GSH structure. Finally, the peak observed at 1520 cm^−1^ is assigned to the N–H bending mode of the peptide bond (amide II), which overlaps with the asymmetrical stretching vibration of the carboxylate anion COO^−^. These bands, which are not present in the spectrum of pure PVC, validate the correct incorporation of GSH onto PVC structure. The ^13^C CP-MAS NMR spectra of PVC, GSH, and PVC-GSH provided further evidence of the successful functionalization of the polymer matrix. The spectrum of PVC is characterized by a single prominent resonance at 54.4 ppm, which corresponds to the carbon atoms of the polymer backbone. The spectrum of GSH exhibits four distinct signals between 32.4 and 55.0 ppm, assigned to the aliphatic carbons of the amino acid residues, as well as intense signal with 3 peaks at 169.4, 173.0 and 177.1 ppm which represents the carbonyl carbons of the carboxyl groups.^45^ The PVC-GSH spectrum reveals signals related to both its constituents. The PVC peak at 54.4 ppm is slightly shifted to 56.3 ppm and undergoes noticeable broadening to the 50–30 ppm zone. This broad band results from the convolution of the main PVC signal with those corresponding to the aliphatic carbons of GSH. The carboxylate signals of pure GSH disappear in PVC-GSH, suggesting a substantial modification of the chemical environment or a change in the relaxation dynamics of the carbonyl groups upon immobilization. Collectively, FTIR and NMR results support the conclusion that GSH was effectively incorporated into the PVC structure. Quantitative bulk elemental analysis was not performed. From the synthesis stoichiometry, the maximum achievable degree of substitution is ∼2.0 mol% of vinyl chloride units (∼0.3 mmol). The XPS surface composition (*S*(*S*^−1^ + Cl) ≈ 1.7 mol%, Section 3.4) indicates a comparable value, corresponding to an estimated GSH loading of ∼0.25 mmol g^−1^. As XPS probes only the outermost few nanometres, this value refers to the near-surface region.

The morphological evolution of the polymer following functionalization was investigated using SEM analysis. As shown in Fig. 1d, PVC particles are characterized by a nearly spherical morphology and a distinct surface roughness. In contrast, the PVC-GSH sample exhibits a loss of this initial uniformity, with particles appearing more elongated in shape. Furthermore, the functionalization with GSH resulted in a more compact structural arrangement compared to the surface texture observed in PVC. These morphological changes are consistent with the modification of the polymer matrix through the incorporation of GSH moieties. Considering the application of PVC-GSH in adsorption experiments, its textural properties were also evaluated by nitrogen adsorption–desorption measurements using the BET method (Fig. S2), which resulted in a specific surface area of 27.6 m^2^ g^−1^ and a total pore volume of 0.033 cm^3^ g^−1^. The isotherm shows low uptake up to *P*/*P*_0_ ≈ 0.85 and a steep rise only at high relative pressure, indicating a low-porosity material with a restricted pore volume.

The thermal properties of PVC-GSH were assessed through TGA (Fig. S3) and DSC (Fig. S4) and compared to those of pristine PVC. TGA analysis revealed that for PVC, the onset of thermal degradation and the associated weight loss began at approximately 230 °C. Following functionalization with GSH, the degradation temperature for PVC-GSH decreased to approximately 170 °C. This reduction in thermal resistance was expected and is directly attributed to the incorporation of GSH, a bio-based molecule with lower thermal stability than the synthetic PVC backbone. Despite this shift, the thermal stability of PVC-GSH remains well within the requirements for environmental remediation and aqueous treatment applications, which typically occur at ambient or slightly higher temperatures. DSC measurements provided further evidence of the successful chemical modification of the polymer. PVC particles exhibited a glass transition temperature (*T*_g_) of 81.8 °C, which increased to 92.1 °C for PVC-GSH (Fig. S4). This shift of approximately 10.3 °C reflects a marked reduction in PVC chain mobility, driven by steric hindrance from the bulky tripeptide moieties and intermolecular hydrogen bonding between the polar groups of GSH.

### 3.2 Adsorption of dyes and effect of pH

The performance of PVC-GSH as a dye adsorbent was first evaluated through an initial screening involving six widely used organic dyes (Fig. 2a). To this end, three anionic dyes (fluorescein, rose Bengal, and methyl orange) and three cationic dyes (crystal violet, rhodamine B, and methylene blue) were selected. As shown in Fig. 2b, a significant uptake of fluorescein, exceeding 62% of the initial amount, was observed. On the other hand, the adsorption of all other tested dyes was negligible, with removal ratios consistently below 5%.

**Fig. 2:**
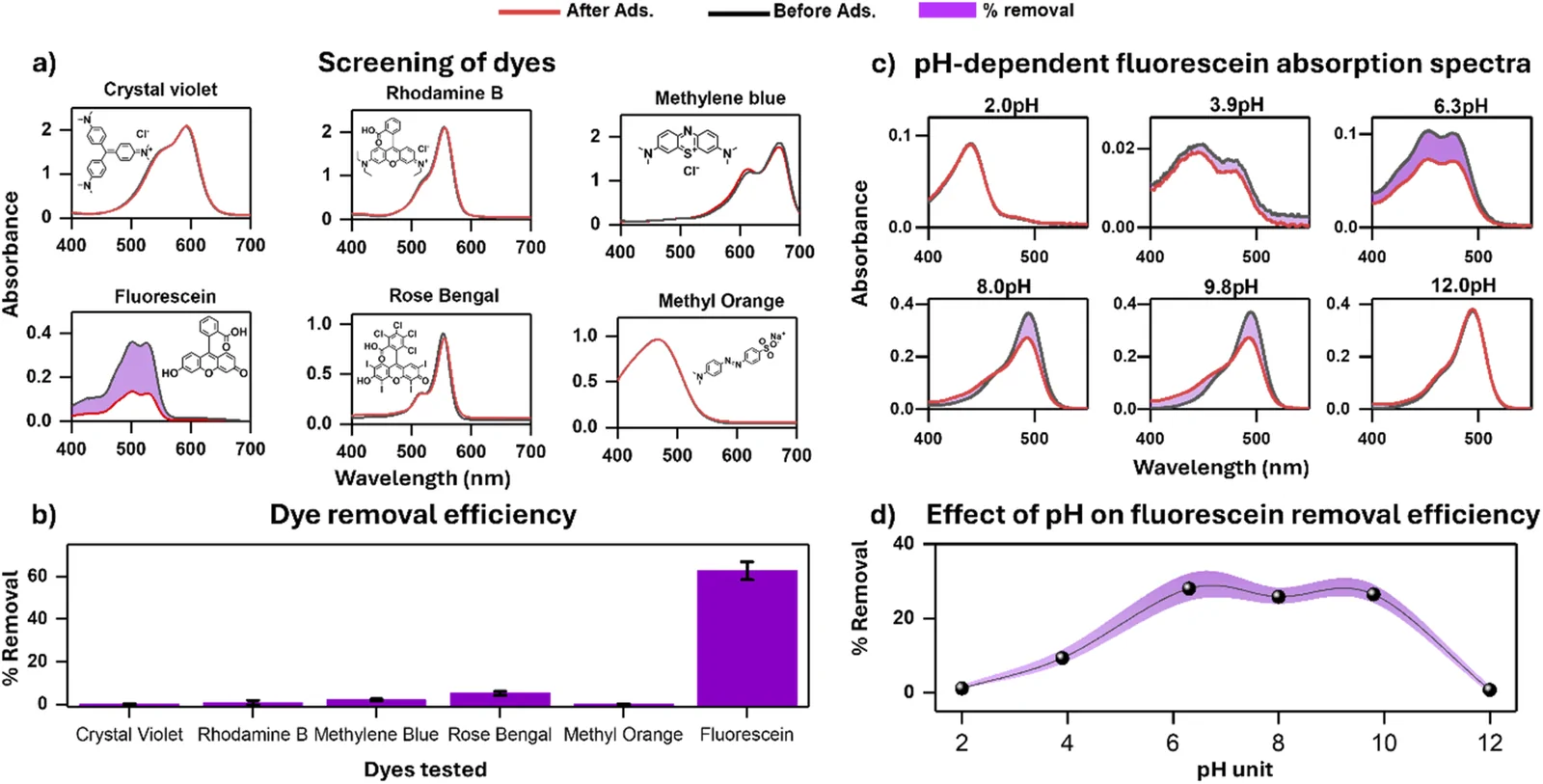
(a) Absorption spectra of 5 mg L^−1^ dye solutions in the presence of 50 mg per L PVC-GSH after 24 h. (b) Removal efficiency (%) of different dyes under the same conditions. (c) Effect of pH on the absorption spectra of a 20 mg L^−1^ fluorescein solution treated with 50 mg per L PVC-GSH for 24 h. (d) Corresponding fluorescein removal (%) as a function of pH.

This pronounced selectivity indicates a specific chemical and/or structural compatibility between PVC-GSH and fluorescein that was not shared with the other dyes. This compatibility can be rationalized on a structural basis. Although rose Bengal shares the xanthene core with fluorescein, its four iodine atoms shield the phenolate and carbonyl oxygens that act as hydrogen-bond acceptors, and its four chlorine atoms hinder access to the carboxylate. In addition, halogenation increases the acidity of the xanthene phenol, so that rose Bengal is fully dianionic at pH 6, whereas fluorescein is present as a monoanion/dianion mixture in which the monoanion retains a phenolic OH group able to act as a hydrogen-bond donor. Methyl orange interacts mainly through a sulfonate group, a weak hydrogen-bond acceptor, and lacks the xanthene/carboxylate moieties. Consequently, subsequent experiments, including pH optimization, kinetic and isotherm modelling, and the mechanistic studies, were focused on understanding the adsorption of fluorescein.

Solution pH is a critical factor governing adsorption efficiency, as it regulates the ionization state of the dye and the surface charge of the adsorbent. As reported in Fig. 2c, fluorescein adsorption was investigated across a broad pH range, from 2 to 12. The adsorption process was highly efficient from 6 to 10, while outside this range the removal of fluorescein sharply declined (Fig. 2d). This behaviour is closely linked to the solution chemistry of fluorescein, which exists in cationic (pH < 2), neutral (2 < pH < 4), monoanionic (4.3 < pH < 6.4), and dianionic (pH > 6.4) forms.^46^ The high efficiency observed between pH 6 and 10 suggests that monoanionic and dianionic species are the primary participants in the adsorption process. Since fluorescein remains dianionic above pH 10, the decrease in uptake at higher pH is more likely related to changes in the adsorbent, such as the deprotonation of the GSH amine groups. To further elucidate the role of surface charge, zeta potential analysis was performed at the optimal condition of pH 6 for PVC-GSH and all tested dyes (Fig. S5 and S6). Interestingly, both PVC-GSH and the fluorescein solution were found to be nearly neutral, with zeta potentials of −4.81 mV and −0.52 mV, respectively. This implies that strong electrostatic interactions are unlikely to be the primary driving force for adsorption at the optimum pH condition. Consistently, the cationic dyes were not adsorbed despite the slightly negative surface of PVC-GSH, confirming that electrostatic attraction alone cannot account for the observed selectivity. Control experiments with unmodified PVC (Fig. S7–S9) showed consistently low removal efficiency (<10%) for all tested dyes at different pH conditions (pH 5 to 9). Therefore, nonspecific adsorption by PVC was negligible and largely independent of pH.

### 3.3 Adsorption kinetic, equilibrium and reusability studies

The practical applicability of PVC-GSH and its physicochemical interactions with fluorescein were investigated through adsorption kinetics, equilibrium isotherms (Fig. 3) and reusability studies.

**Fig. 3:**
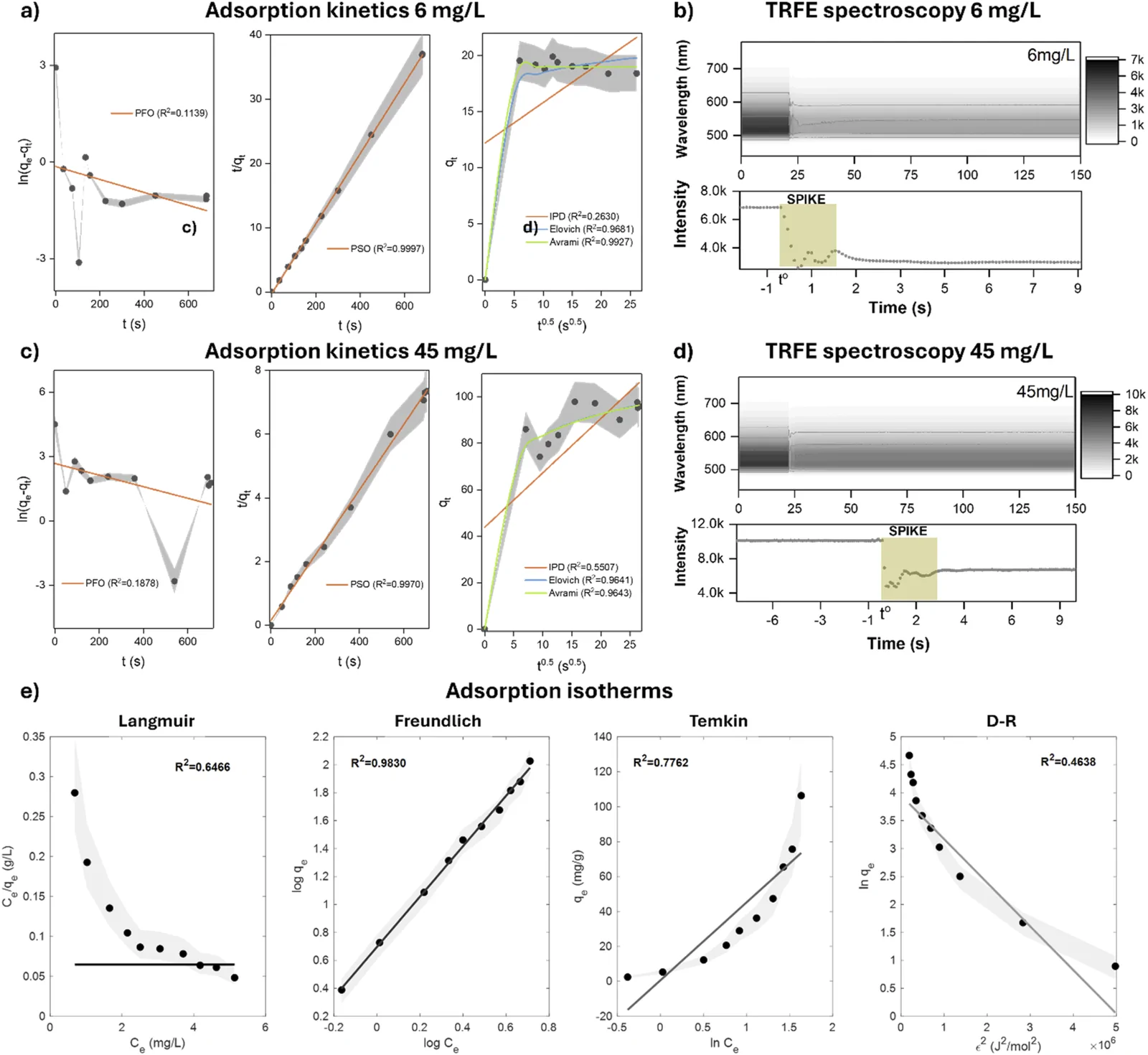
Kinetic modelling of adsorption data obtained *via* UVvis spectroscopy for 6 mg L^−1^ (a) and 45 mg L^−1^ (c) fluorescein solutions treated with PVC-GSH (50 mg L^−1^) at pH 6, showing fits to PFO, PSO, IPD, Elovich, and Avrami models. TRFE spectroscopy measurements for 6 mg L^−1^ (b) and 45 mg L^−1^ (d) fluorescein solutions, displaying emission wavelength–time intensity maps and corresponding temporal intensity traces highlighting the injection (spike) region. (e) Equilibrium adsorption isotherms for fluorescein removal with PVC-GSH (50 mg L^−1^) at pH 6 and room temperature, fitted using Langmuir, Freundlich, Temkin, and D–R models.

Initially, kinetic studies were performed using conventional UV-vis spectroscopy by monitoring the fluorescein adsorption over time. This first investigation showed that the adsorption process was completed within the timescale limitation of our set-up (∼30 s) at both low (6 mg L^−1^, Fig. 3a and S10) and high (45 mg L^−1^, Fig. 3c) concentrations of fluorescein.

To evaluate the kinetic data, the experimental points were fitted to five linearized kinetic models, including PFO, PSO, Elovich, IPD, and Avrami (Table S1). Although the PSO model yields very high correlation coefficients (*R*^2^ > 0.99), this is a mathematical consequence of *q*_*t*_ ≈ *q*_e_ across all measured time points rather than evidence of a specific mechanism. Because the actual uptake rate exceeds the temporal resolution of our standard batch UV-vis protocol, these linearized kinetic models cannot discriminate between underlying adsorption mechanisms or rate-limiting steps. Consequently, the fitting parameters are reported in Table S1 for descriptive completeness only, without drawing mechanistic conclusions.

To overcome the timescale limitation of our UV-vis setup, the study was refined by analysing the adsorption of 6 mg L^−1^ (Fig. 3b) and 45 mg L^−1^ (Fig. 3d) fluorescein solutions on the ms timescale *via* TRFE spectroscopy. From these measurements, we observed that following the initial addition of the adsorbent, the fluorescence signal reached a plateau at a 60% reduction within ∼3 s at both concentrations. Since this timescale is comparable to the mixing time in the stirred cuvette, it represents an upper limit for the adsorption timescale and is likely governed by mixing and external mass transfer, not by the intrinsic binding steps. Because inner-filter effects, particle scattering and possible quenching of adsorbed fluorescein can contribute to the fluorescence signal, the TRFE data were used only to determine the timescale of the process, and adsorbed amounts were quantified exclusively from supernatant UV-vis measurements.

To investigate the interaction between PVC-GSH and fluorescein at equilibrium, the experimental data were fitted using four widely utilized models: Langmuir, Freundlich, Temkin, and D–R (Fig. 3e). These models can provide information on surface homogeneity, the nature of the binding sites, and whether the process follows a monolayer or multilayer mechanism. The calculated parameters for each model are summarized in Table S2. The Langmuir model yielded a poor correlation (*R*^2^ = 0.6466) and physically unrealistic parameters, such as a theoretical maximum capacity *q*_m_ of the order of 10^6^ mg g^−1^. This mathematical divergence, visible in the downward curvature of the *C*_e_/*q*_e_*vs. C*_e_ plot, confirms that the adsorption is not restricted to a simple, homogeneous monolayer. Similarly, both the Temkin (*R*^2^ = 0.7762) and D–R (*R*^2^ = 0.4638) models had a weak fit of the adsorption data, suggesting that neither a linear decrease in adsorption heat nor a simple pore-filling mechanism could describe the system. The data were best described by the empirical Freundlich model (*R*^2^ = 0.983), suggesting cooperative adsorption in which adsorbed fluorescein facilitates further uptake, possibly through π–π interactions between xanthene units. Although PVC-GSH did not reach full saturation within the experimental concentration range, the highest experimentally observed uptake was ∼97 mg g^−1^. To contextualize this performance, PVC-GSH was compared with recently reported adsorbents for fluorescein removal. The adsorption capacity of PVC-GSH compares well with several established materials, including herb-based microparticles^47^ (2.6–2.9 mg g^−1^ in 16 h), Cu-isonicotinate MOFs^48^ (66.7 mg g^−1^ in 6 h), and aminosilica particles^49^ (132 mg g^−1^ in 24 h). While certain advanced materials, such as Al/Th MOFs, exhibit a higher maximum capacity of 668.8 mg g^−1^,^50^ it is noteworthy that these systems require equilibrium times ranging from 1 to 24 hours. This duration is significantly longer than the fast kinetic profile of PVC-GSH, for which uptake was complete within ∼30 s by UV-vis, with a signal change within ∼3 s in the TRFE measurements.

The reusability of PVC-GSH was evaluated over four adsorption–desorption cycles. Owing to the negligible fluorescein uptake at pH 12 (Fig. 2d), 10^−2^ M NaOH was used as eluent. PVC-GSH retained 87.1% of its initial removal efficiency in the 2nd cycle and 64.1% in the 4th (Fig. S11a). The stability of the grafted GSH was also tested by stirring at 200 rpm PVC-GSH for 24 h in the aqueous matrix used for the adsorption experiments. The FTIR spectrum recorded afterwards was unchanged, with the GSH-related bands at 1742, 1664 and 1520 cm^−1^ fully retained (Fig. S11b).

### 3.4 Adsorption mechanism

To elucidate the binding mechanism between fluorescein and PVC-GSH, a spectroscopic investigation was conducted using FTIR and XPS analyses (Fig. 4).

**Fig. 4:**
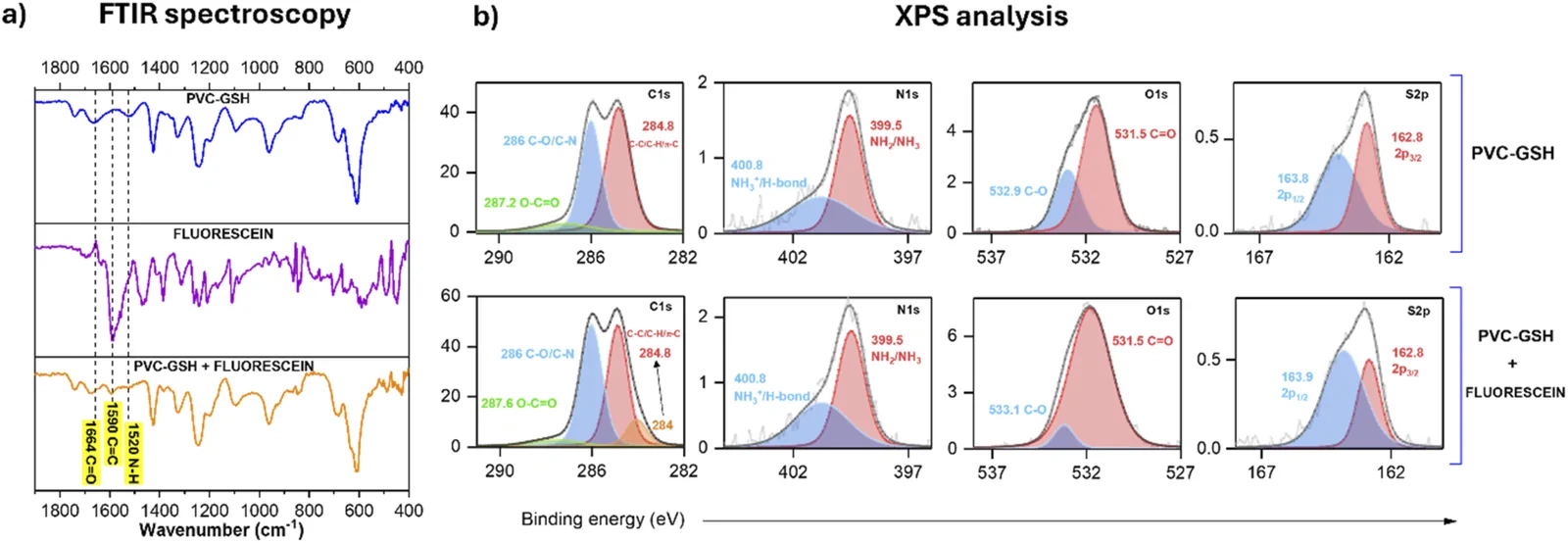
(a) FTIR spectra of PVC-GSH, fluorescein, and PVC-GSH after the adsorption of fluorescein. (b) XPS spectra of PVC-GSH before and after adsorption of fluorescein.

The FTIR spectra of PVC-GSH before and after adsorption were compared with the spectrum of pure fluorescein to identify the functional groups involved in the process (Fig. 4a). The characteristic bands of PVC-GSH, specifically the C=O stretching (amide I) at 1664 cm^−1^ and the N–H bending (amide II) at 1520 cm^−1^, were significantly affected by the adsorption of the dye. After interaction, the amide I peak shifted toward higher wavenumbers, while the relative intensity of the amide II band notably decreased. These changes suggest that the carboxyl and amino/amide groups of the GSH moieties are involved in binding. The observed shifts are consistent with the formation of a complex network of hydrogen bonds and/or dipole–dipole interactions between the GSH functional groups and the xanthene or carboxylate groups of fluorescein. Furthermore, the spectrum of PVC-GSH after adsorption showed the clear emergence of characteristic fluorescein bands, such as the intense C=C aromatic stretching band at 1590 cm^−1^.

To obtain additional evidence of the binding mechanism, XPS analysis was performed before and after the adsorption of fluorescein with PVC-GSH (Fig. 4b). The relative concentrations of elements in XPS spectra (Table S3) confirmed the presence of fluorescein on the surface, marked by a 51.4% relative increase in O 1s concentration and a simultaneous decrease of 2.55 at% (8.3% relative) in the Cl 2p signal from the underlying PVC substrate. This “masking effect” indicates the formation of an organic overlayer that attenuates the photoelectrons escaping from the PVC backbone. Notably, the N 1s concentration remained constant and the S 2p signal decreased only slightly, in proportion to the attenuation of the Cl 2p signal, suggesting that GSH remains tethered to the surface during dye adsorption. High-resolution analysis provided deeper mechanistic insights. In the C 1s region (Table S4), the C–O/C–N component at 286.0 eV increased by ∼8%, consistent with the oxygen-rich xanthene structure of fluorescein. Even more diagnostic was the O 1s region (Table S5), where the component at 533.1 eV (representing phenolic and carboxylate oxygens) exhibited a 5.2-fold increase in area. This specific oxygen signature serves as primary chemical evidence for fluorescein capture. Regarding the nitrogen environment (Table S6), while the total N 1s concentration remained constant, the fraction of protonated or H-bonded nitrogen (400.8 eV) grew from 45.53% to 54.98%. This shift suggests ion pairing and/or hydrogen bonding between fluorescein and the GSH amine/amide groups, consistent with the FTIR observations. Furthermore, the S 2p analysis (Table S7) confirmed that sulfur remains entirely in its reduced state (162.8 eV), with no evidence of oxidation during the process. Based on the Cl 2p data (Fig. S12), there is no evidence of degradation (like dechlorination) or chemical cross-linking occurring at the chlorine-containing positions of the polymer. In addition, to quantify the physical scale of the adsorption, the attenuation of the Cl 2p backbone marker (Table S8) was modeled using the Beer–Lambert equation.^51^ By treating the PVC as a semi-infinite substrate beneath a uniform organic layer, assuming an inelastic mean free path between 2.5 and 3.5 nm,^52,53^ and using the survey atomic percentages (Table S9) as a proxy for total peak intensity, the overlayer thickness was estimated to be 0.22–0.30 nm. As this is smaller than the thickness of a flat-lying aromatic layer, it indicates sub-monolayer average coverage at the outermost surface at the dye loading analysed, and the absence of thick dye aggregates. This is consistent with fluorescein bound at GSH-functionalized surface sites, in agreement with the FTIR and N 1s changes discussed above. The XPS data alone, however, do not establish the orientation of the adsorbed molecules or the nature of the binding interactions, which, in addition to hydrogen bonding, can also involve ion pairing, π–π stacking between xanthene units and hydrophobic interactions with the PVC backbone.

### 3.5 Antioxidant properties of PVC-GSH

The antioxidant properties of PVC-GSH were determined and compared with those of PVC through the DPPH free radical scavenging assay. This assay, first introduced by Blois^54^ and then extensively refined in the following decades, represents one of the most straightforward methods to determine the antioxidant activity of novel materials.^55^ As shown in Fig. 5, unmodified PVC exhibited negligible antioxidant activity across the entire tested concentration range (200–1800 mg L^−1^). The inhibition ratio remained nearly constant at approximately 11% (within the experimental error margin), indicating that pristine PVC possesses no intrinsic radical scavenging properties. In contrast, PVC-GSH demonstrated a significant concentration-dependent increase in antioxidant activity. Specifically, the inhibition ratio rose sharply from 10% at a concentration of 200 mg L^−1^ to 80% at 1000 mg L^−1^, finally reaching a ∼95% inhibition at 1800 mg L^−1^.

**Fig. 5:**
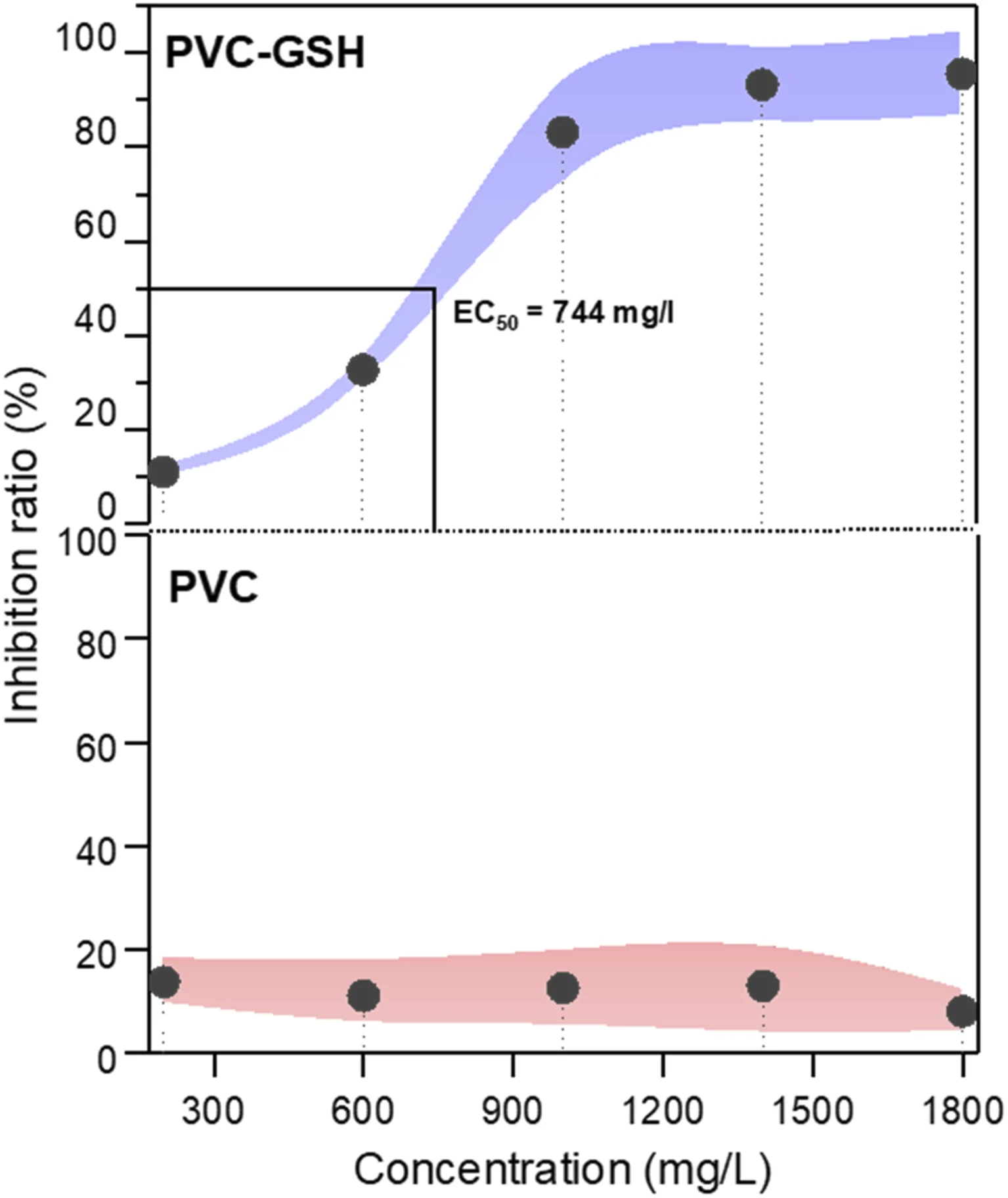
Evaluation of the radical scavenging activity of PVC-GSH and PVC against 10 µM DPPH solution at polymer concentrations ranging from 200 to 1800 mg L^−1^.

The enhancement in antioxidant activity observed for PVC-GSH compared to pristine PVC is attributed to the incorporation of GSH molecules, which serve as active centres for radical neutralization.^56^ The amount of substrate required to scavenge 50% of the DPPH radicals, defined as the half-maximal effective concentration (EC_50_, often referred to as IC_50_),^57,58^ was determined to be 774 mg L^−1^ for PVC-GSH. Normalizing this value to the grafted GSH content (∼0.25 mmol g^−1^ from XPS and ∼0.30 mmol g^−1^ as stoichiometric limit, Section 3.1) corresponds to 59–71 mg per L GSH equivalents. This is close to the EC_50_ values reported for free GSH^59,60^ (70–81 mg L^−1^, Table 1), indicating that a substantial part of the radical-scavenging capacity of GSH is retained after immobilization. When compared to other polymeric scavengers reported in the literature (Table 1), the EC_50_ of PVC-GSH falls within an intermediate range of values.

**Table 1:** DPPH radical scavenging activities (EC_50_ values) of free GSH and of polymeric materials reported in the literature, with the initial DPPH concentration and reaction time of each assay^a^

Material	Initial DPPH concentration (mM)	Time (min)	EC_50_ (mg L^−1^)
Chitosan-conjugated phytocompounds^61^	1	60	12
Free GSH^59,60^	0.2; not reported	20; 30	70–81
Phloroglucinol-encapsulated starch biopolymer^62^	0.065	30	120
PVC-GSH	0.1	15	774
Chrysin and piperine in chitosan and lecithin nanoparticles^63^	Not reported	30	1000
Agaro-oligosaccharides^64^	0.15	30	1850–8000
GP70-2 (d-galactose and l-arabinose (1 : 1))^65^	0.2	30	10 800
Levan polymer^66^	Not reported	30	24 420

a As the DPPH EC_50_ depends on the assay conditions, which vary among the cited studies, and DPPH scavenging is only one indicator of antioxidant capacity, these comparisons should be regarded as indicative.

## 4 Conclusions

The synthesis of PVC-GSH demonstrates a potential strategy for the valorization and upcycling of PVC into a multifunctional material for environmental applications. Unlike traditional adsorbents, this material exploits the inherent bio-activity of glutathione (GSH), transforming a common and inert polymer like PVC into a functional material for the rapid removal of organic dyes. The novelty of this work lies in the synergy between the chemical robustness of the PVC backbone and the extended network of chelating sites provided by the tripeptide moieties of GSH.

PVC-GSH exhibits optimal adsorption performance at pH 6.0, and the equilibrium data are best described by an empirical Freundlich isotherm, reaching the highest experimentally observed uptake of ∼97 mg g^−1^. Post-adsorption analyses *via* FTIR and XPS indicate that binding occurs likely *via* hydrogen bonding involving the GSH amino and amide groups, alongside possible contributions from other non-covalent interactions. Fluorescein uptake is very fast, with a signal change within ∼3 s, a timescale limited by mixing, whereas most fluorescein adsorbents reported in the recent literature require hours to reach equilibrium. The fast uptake, coupled with high selectivity, opens interesting scenarios for the development of chemical sensors and real-time monitoring devices, where fast response is critical. Future research will focus on evaluating the performance of PVC-GSH in real water matrices, assessing the effects of competitive adsorption in multi-component dye mixtures and natural organic matter, as well as testing long-term adsorbent stability.

Beyond its use as an adsorbent, PVC-GSH retains part of the GSH radical scavenging ability, as indicated by the DPPH assay, and can therefore act as a dual-function bioactive material. Its EC_50_ value of 774 mg L^−1^ is comparable to that of free GSH on a molar basis, suggesting that a substantial part of the GSH moieties remain active after grafting onto PVC.

## Author contributions

A. Giove: writing – original draft, writing – review & editing, visualization, methodology, investigation, formal analysis, data curation, conceptualization. A. Raj: writing – original draft, writing – review & editing, visualization, methodology, investigation, formal analysis, data curation. N. Chelampetty Para: visualization, investigation, formal analysis. M. Haukka: writing – review & editing, visualization, supervision, resources, funding acquisition, project administration, conceptualization.

## Conflicts of interest

There are no conflicts to declare.

## Supplementary Material

RA-OLF-D6RA07765J-s001

## Data Availability

The data supporting this article have been included in the main text and as part of the supplementary information (SI). Supplementary information is available. See DOI: https://doi.org/10.1039/d6ra07765j.
