# Supplementary material for: Glutathione-functionalized PVC for hydrogen-bonding assisted fluorescein adsorption and radical scavenging
Source: RSC Adv. 2026 Oct 30. Online ahead of print. doi: 10.1039/d6ra07765j (PMC13721795; doi:10.1039/d6ra07765j)
Supplement: Supplementary file 1 [file RA-OLF-D6RA07765J-s001.pdf]

Supplementary Information (SI) for RSC Advances.
This journal is © The Royal Society of Chemistry

Glutathione-Functionalized PVC for Hydrogen-Bonding Assisted Fluorescein Adsorption and Radical Scavenging

Alessio Giove*[a], Amar Raj [a,b], Nihal Chelampetty Para[a] and Matti Haukka*[a]

[a.] University of Jyväskylä, Department of Chemistry, 40014 Jyväskylä, Finland. Email: alessio.a.giove@jyu.fi, matti.o.haukka@jyu.fi

[b.] University of Jyväskylä, Nanoscience Center, 40014 Jyväskylä, Finland

SUPPLEMENTARY INFORMATION

Calibration curve of fluorescein

Fluorescein concentration was determined by UV–Vis spectrophotometry at 490 nm using a calibration curve in the 0–50 mg L^{-1} concentration range (R^2 = 0.993).

The calibration data were fitted using a nonlinear model of the form $y = Ax/(B + x)$, where $A = 0.87 \pm 0.04$ and $B = 23.15 \pm 1.80$.

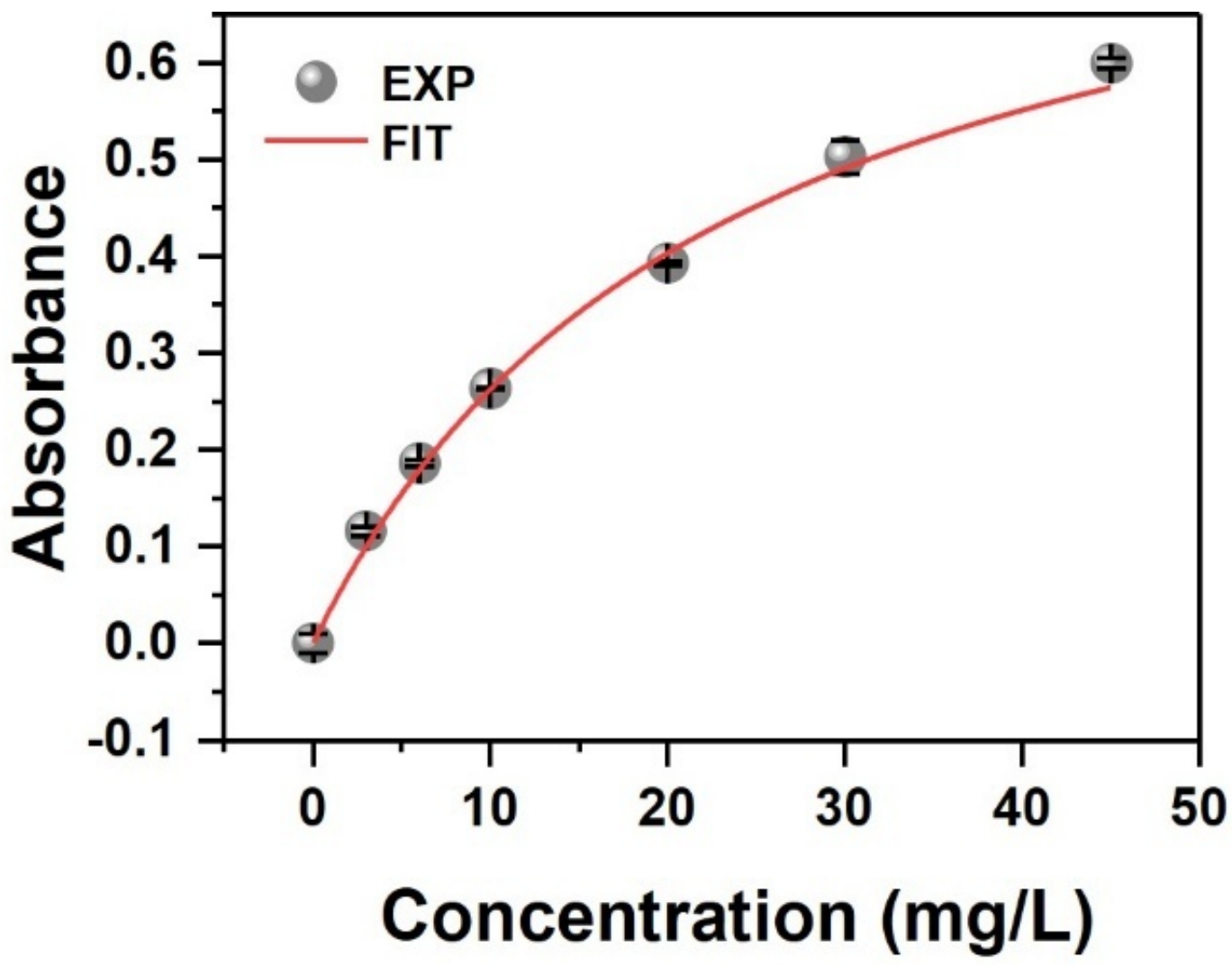

Figure S1. Calibration curve of fluorescein.

Nitrogen adsorption-desorption experiments

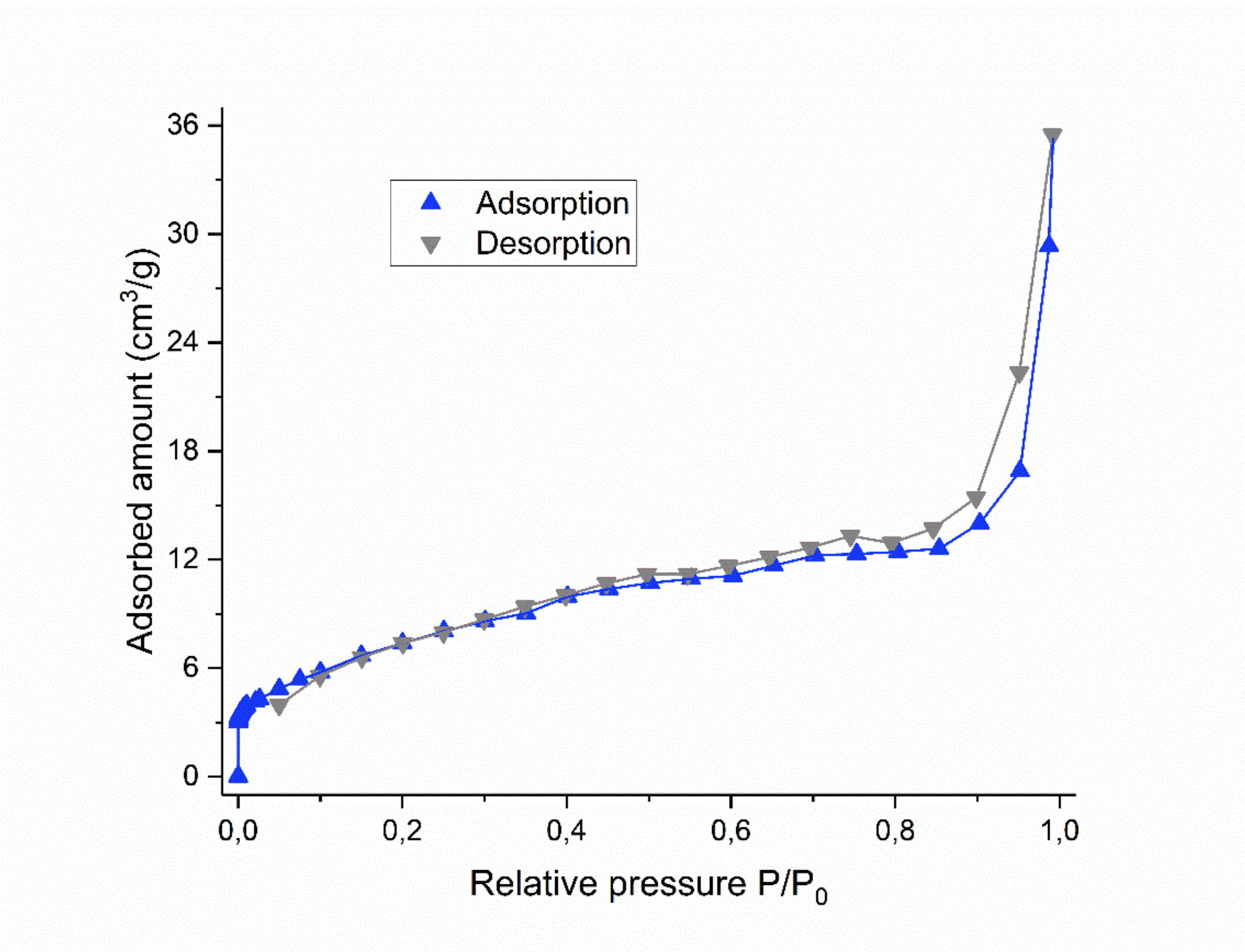

Figure S2. Nitrogen adsorption-desorption isotherms of PVC-GSH polymer particles.

Thermogravimetric analysis

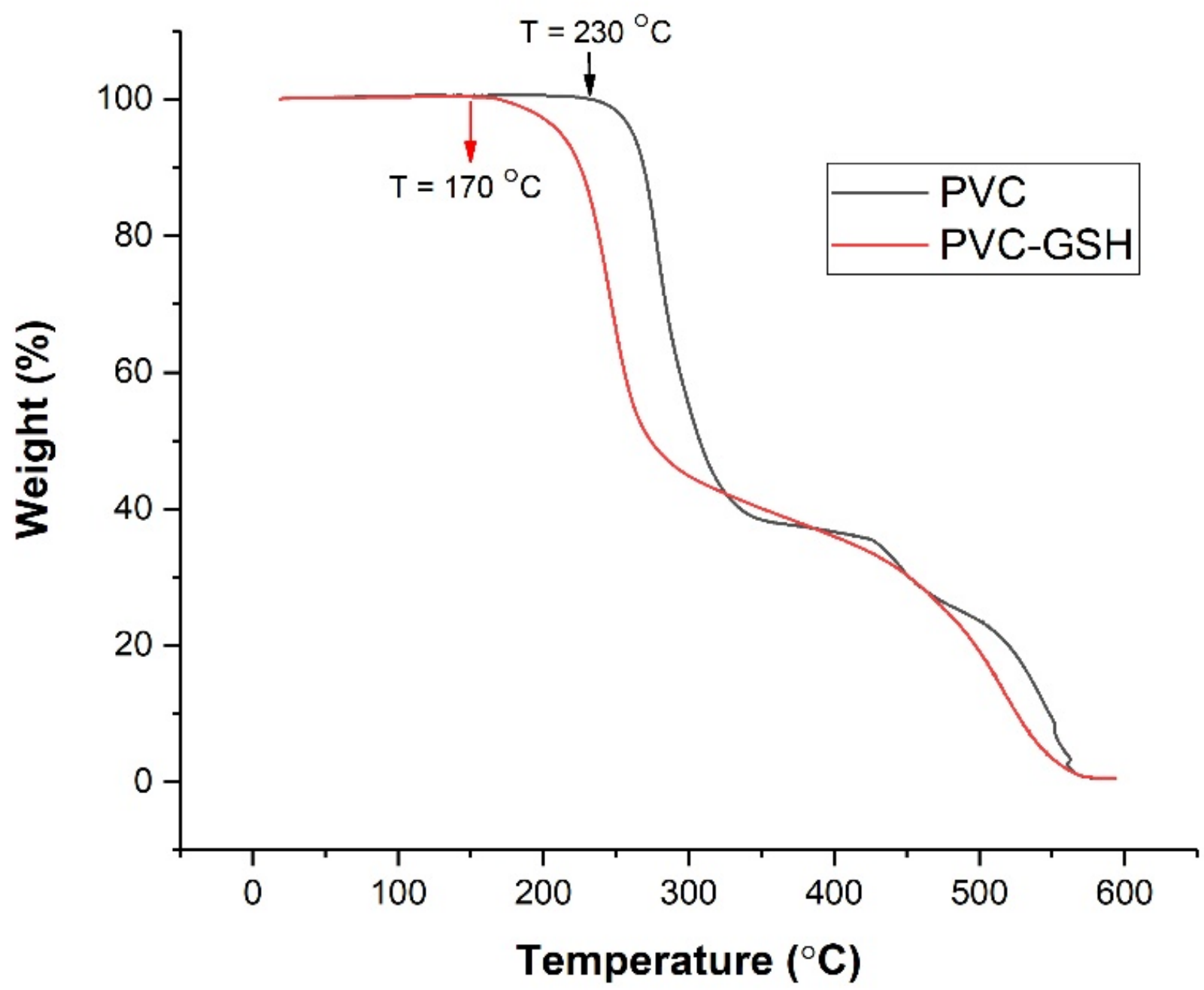

Figure S3. TGA curves of PVC and PVC-GSH polymer particles.

Differential scanning calorimetry

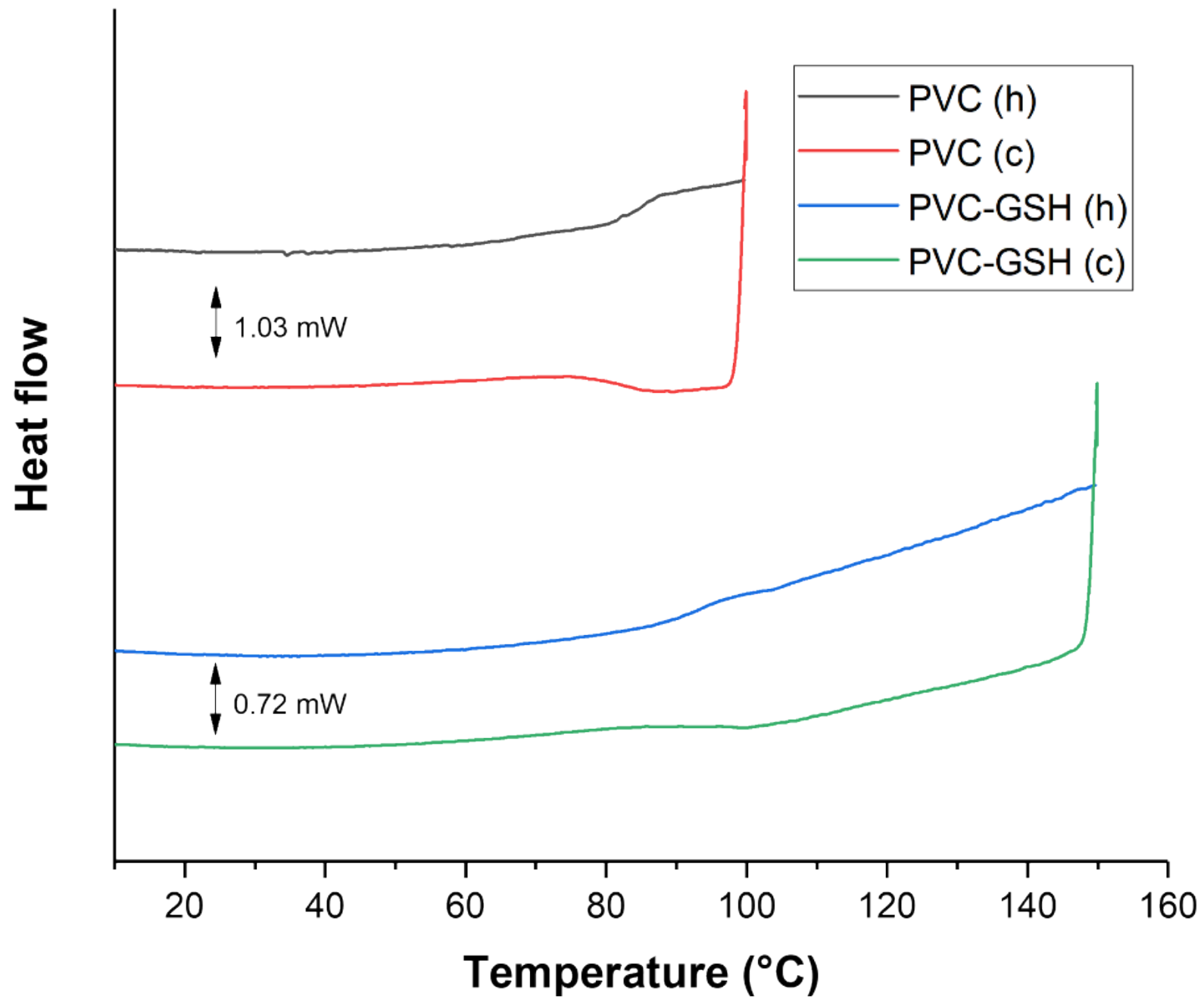

Figure S4. Heating (h) and cooling (c) DSC curves of PVC and PVC-GSH polymer particles.

Zeta Potential

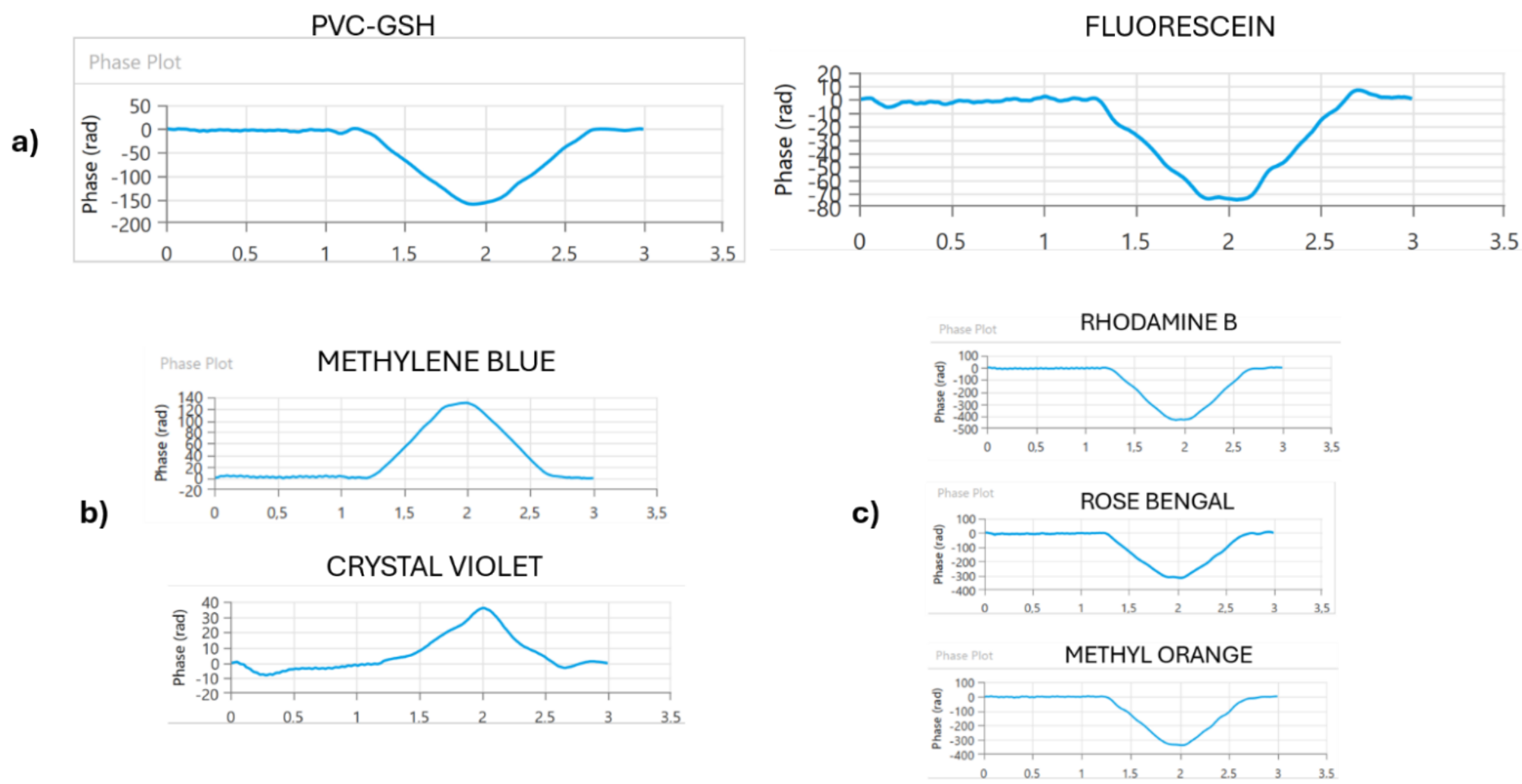

Figure S5. Phase plots of PVC-GSH polymer particles and dyes.

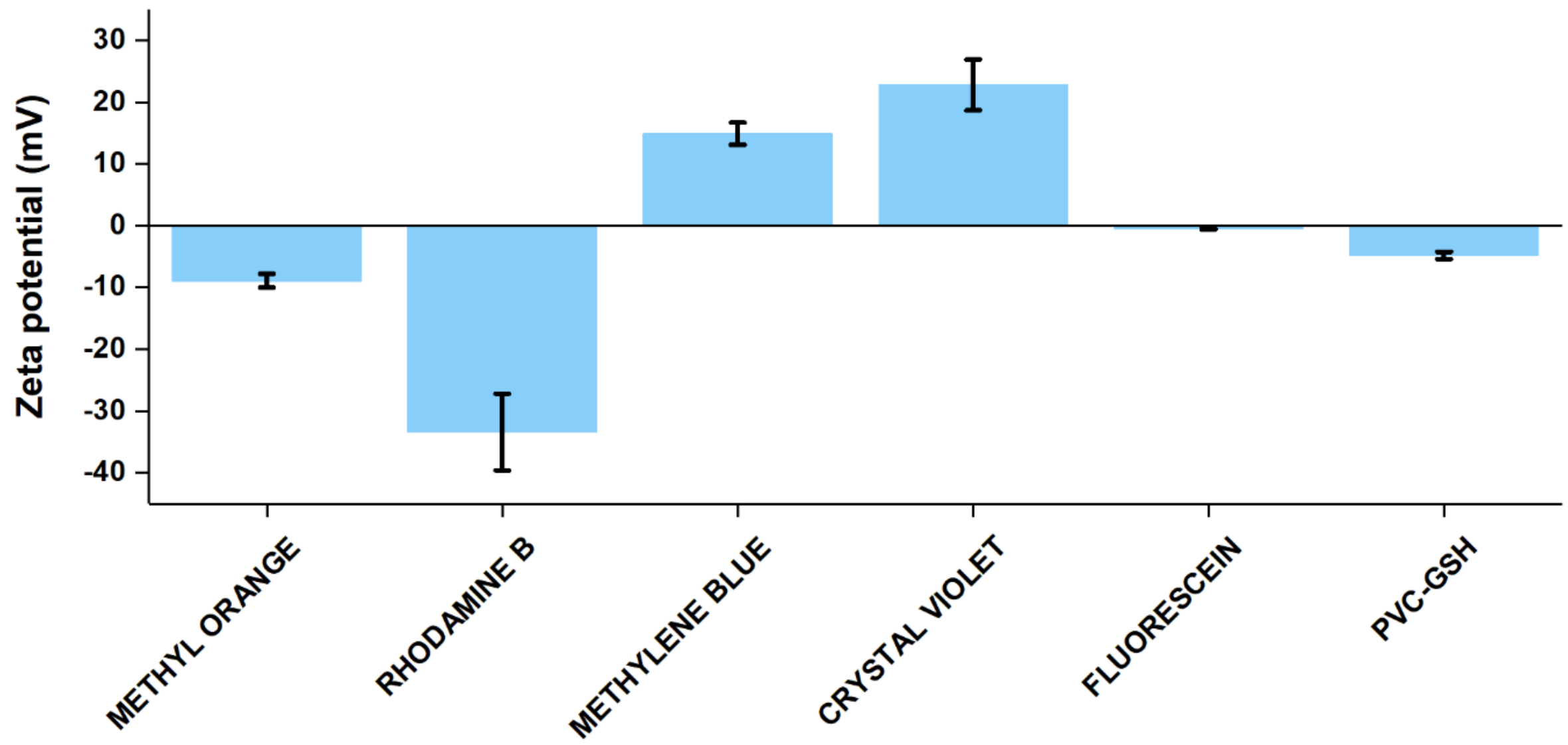

Figure S6. Zeta potential of PVC-GSH polymer particles and dyes.

Control adsorption experiments with unmodified PVC at pH 5-9

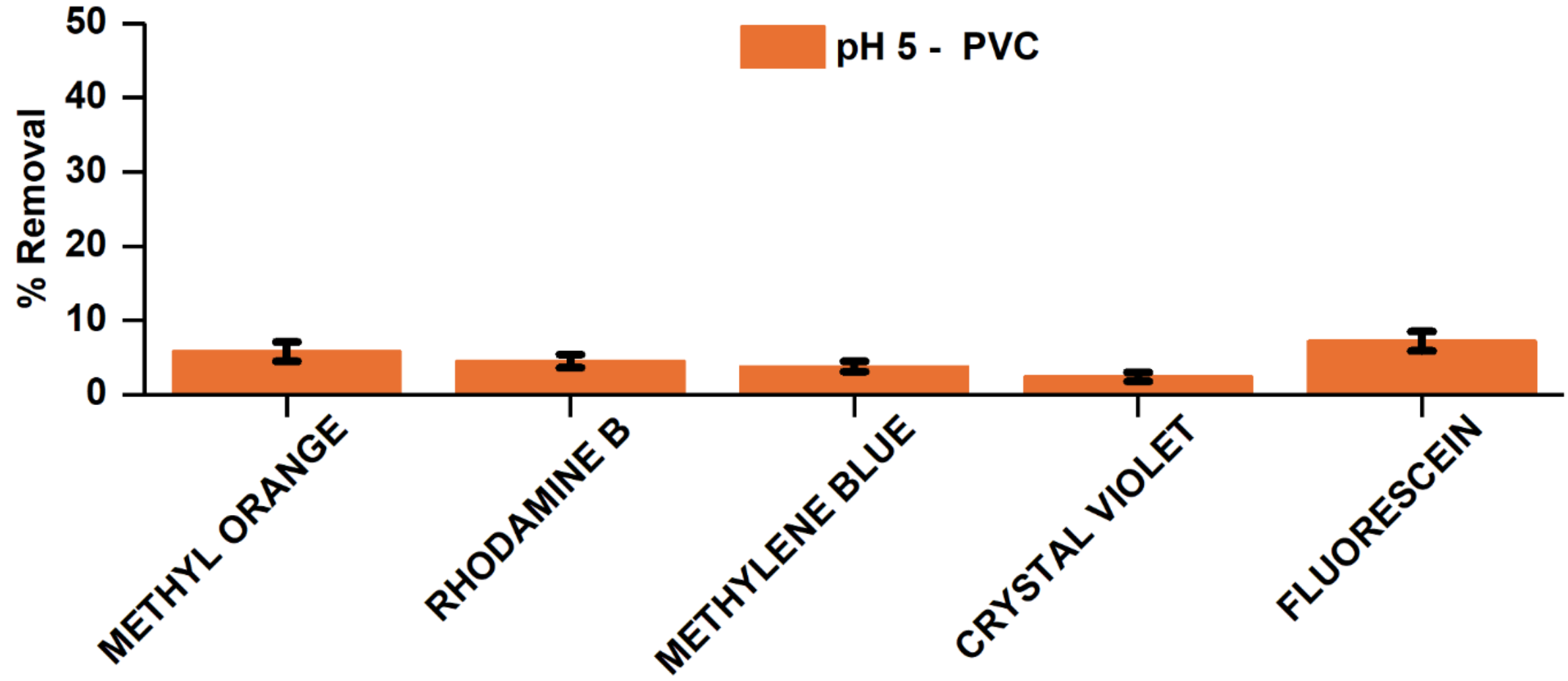

Figure S7. Control adsorption measurements for unmodified PVC. Dye solutions (5 mg/L) were exposed to 50 mg/L PVC for 24 h at pH 5. Dye removal (%) was used to assess nonspecific adsorption of dyes by PVC.

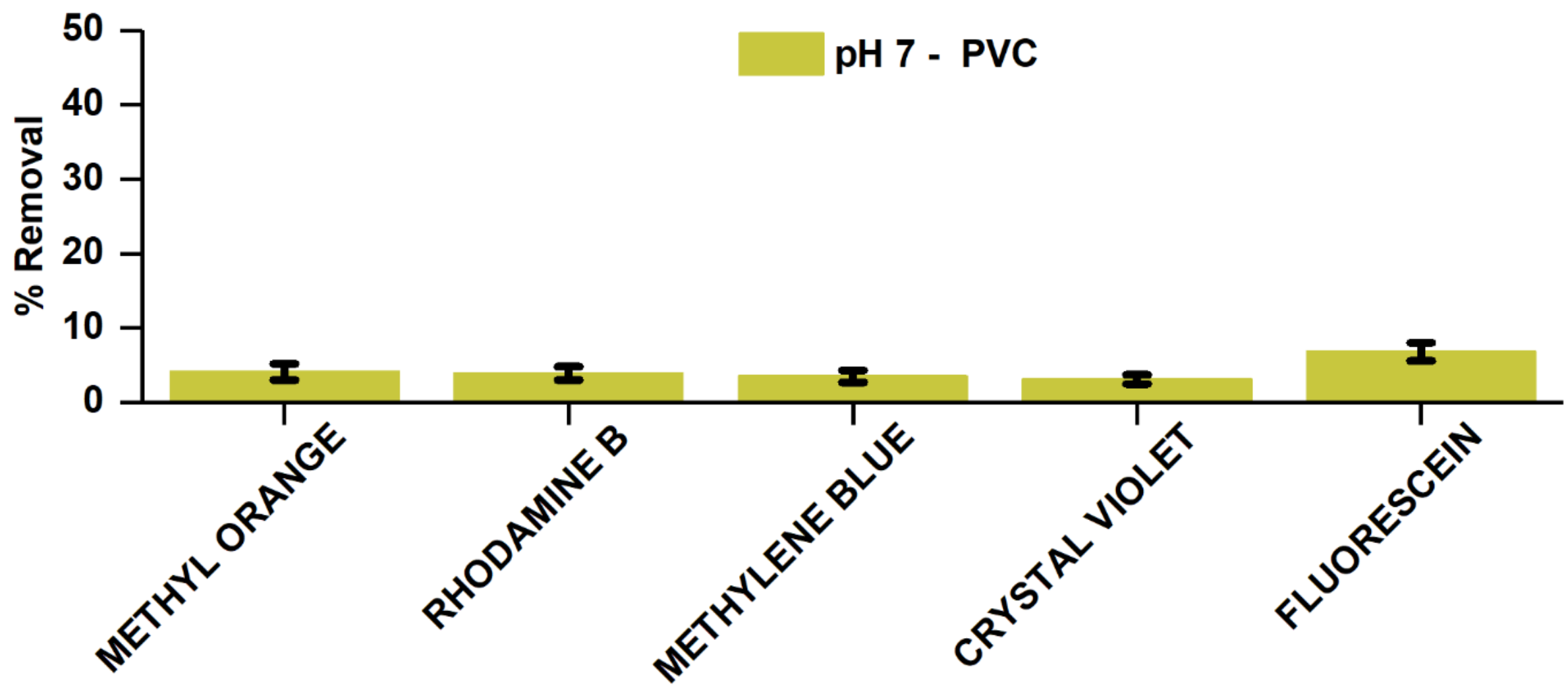

Figure S8. Control adsorption measurements for unmodified PVC. Dye solutions (5 mg/L) were exposed to 50 mg/L PVC for 24 h at pH 7. Dye removal (%) was used to assess nonspecific adsorption of dyes by PVC.

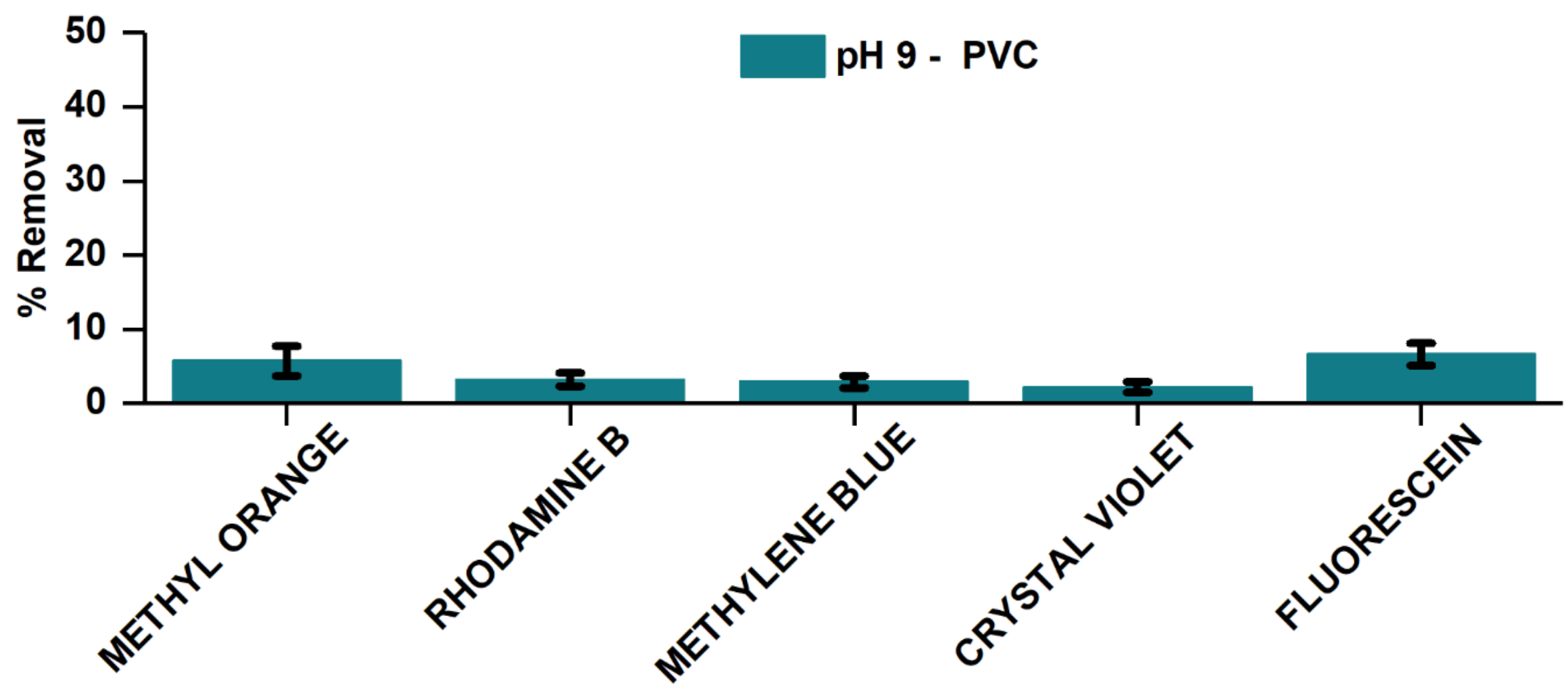

Figure S9. Control adsorption measurements for unmodified PVC. Dye solutions (5 mg/L) were exposed to 50 mg/L PVC for 24 h at pH 9. Dye removal (%) was used to assess nonspecific adsorption of dyes by PVC.

Adsorption kinetic

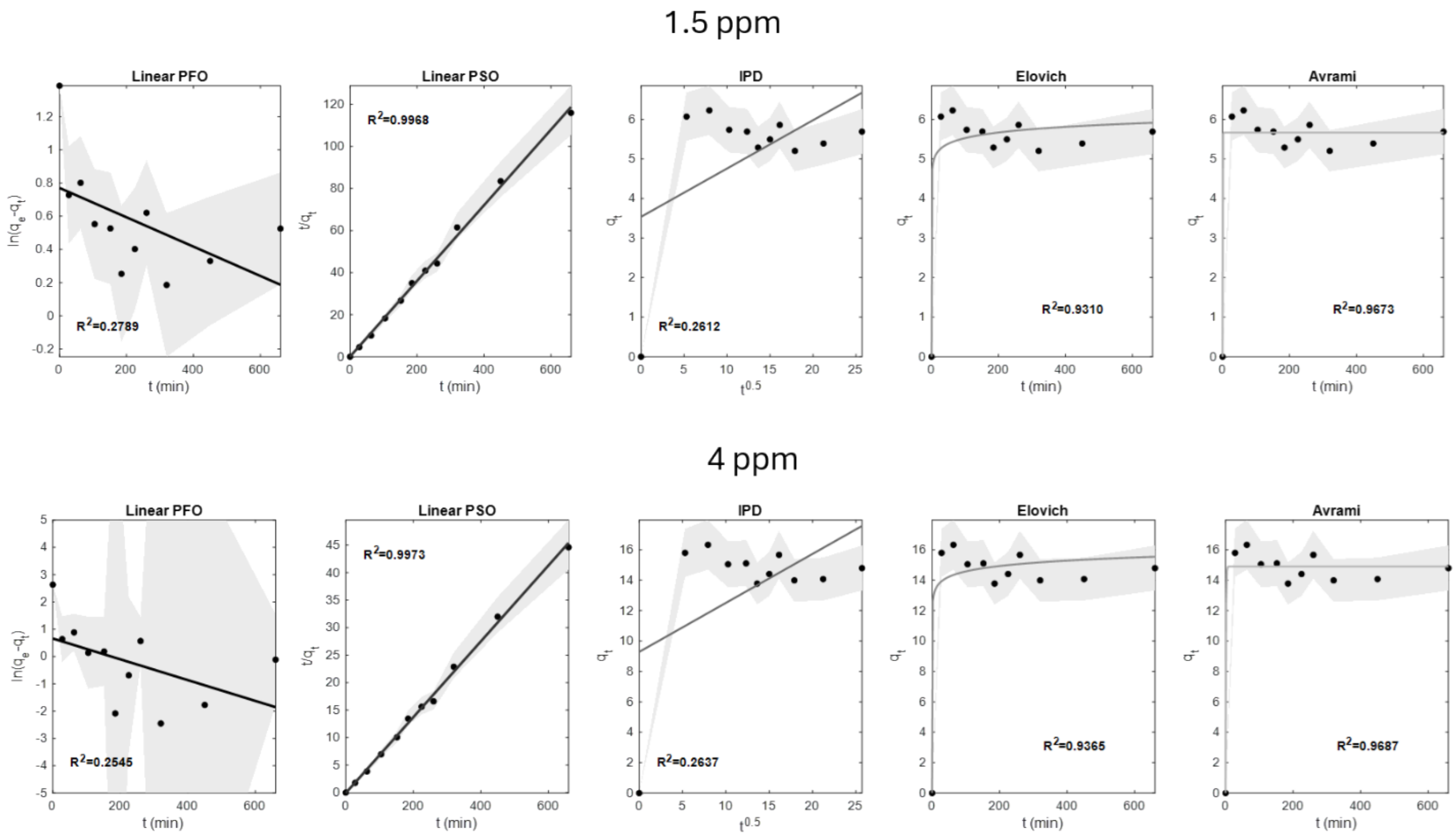

Figure S10. Kinetic modelling of adsorption data obtained via UV–Vis spectroscopy for 1.5 mg/L and 4 mg/L fluorescein solutions treated with PVC-GSH (50 mg/L) at pH 6, showing fits to PFO, PSO, IPD, Elovich, and Avrami models.

Table S1. Kinetic parameters for the adsorption of 1.5 to 45 mg/L fluorescein solutions onto PVC-GSH polymer particles.

Model	Parameter	1.5 mg/L	4 mg/L	6 mg/L	45 mg/L
PFO	k_1 (min⁻¹)	-0.0008	-0.0038	-0.0020	-0.0027
	q_e (mg/g)	2.16	1.93	0.86	14.47
	R^2	0.2788	0.2545	0.1139	0.1878
PSO	k_2 (g mg⁻¹ min⁻¹)	0.1798	0.0690	0.0546	0.0103
	q_e (mg/g)	5.56	14.50	18.31	97.45
	R^2	0.9968	0.9973	0.9997	0.9970
Elovich	α (mg g⁻¹ min⁻¹)	$1.06 * 10^9$	$1.34*10^{10}$	1.04×10^{10}	1.89×10^4
	β (g mg⁻¹)	0.205	0.510	0.659	6.641
	R^2	0.9310	0.9365	0.9681	0.9641
IPD	k_{id} (mg g⁻¹ min□·□)	2.2153	0.6381	0.6047	0.0001
	C (mg/g)	1.0481	0.9917	1.0839	0.1071

	R^2	0.2612	0.2637	0.2630	0.5507
Avrami	k_{av} (min⁻^{1})	0.1222	0.3224	0.3610	2.3415
	n	3.54	9.28	12.21	43.88
	R^2	0.9673	0.9961	0.9927	0.9643
**Experimental**	$q_{e,exp}$ **(mg/g)**	**5.69**	**14.50**	**18.34**	**95.90**

Adsorption isotherms

Table S2. Isotherm model parameters for fluorescein adsorption onto PVC-GSH polymer particles.

**Isotherm Model**	**Parameter**	**Value**	R^2
**Langmuir**	q_m (mg/g)	996,879.60	0.6466
	K_L (L/mg)	0.0000	
	Rᴸ	0.9996	
**Freundlich**	K_F (mg/g)(L/mg)$^{(1/n)}$	4.97	0.9830
	n_F	0.55	
**Temkin**	A (L/mg)	1.01	0.7762
	b_T (J/mol)	55.57	
**D–R**	q_m (mg/g)	52.17	0.4638
	ε (J/mol)	799.97	

Reusability and stability

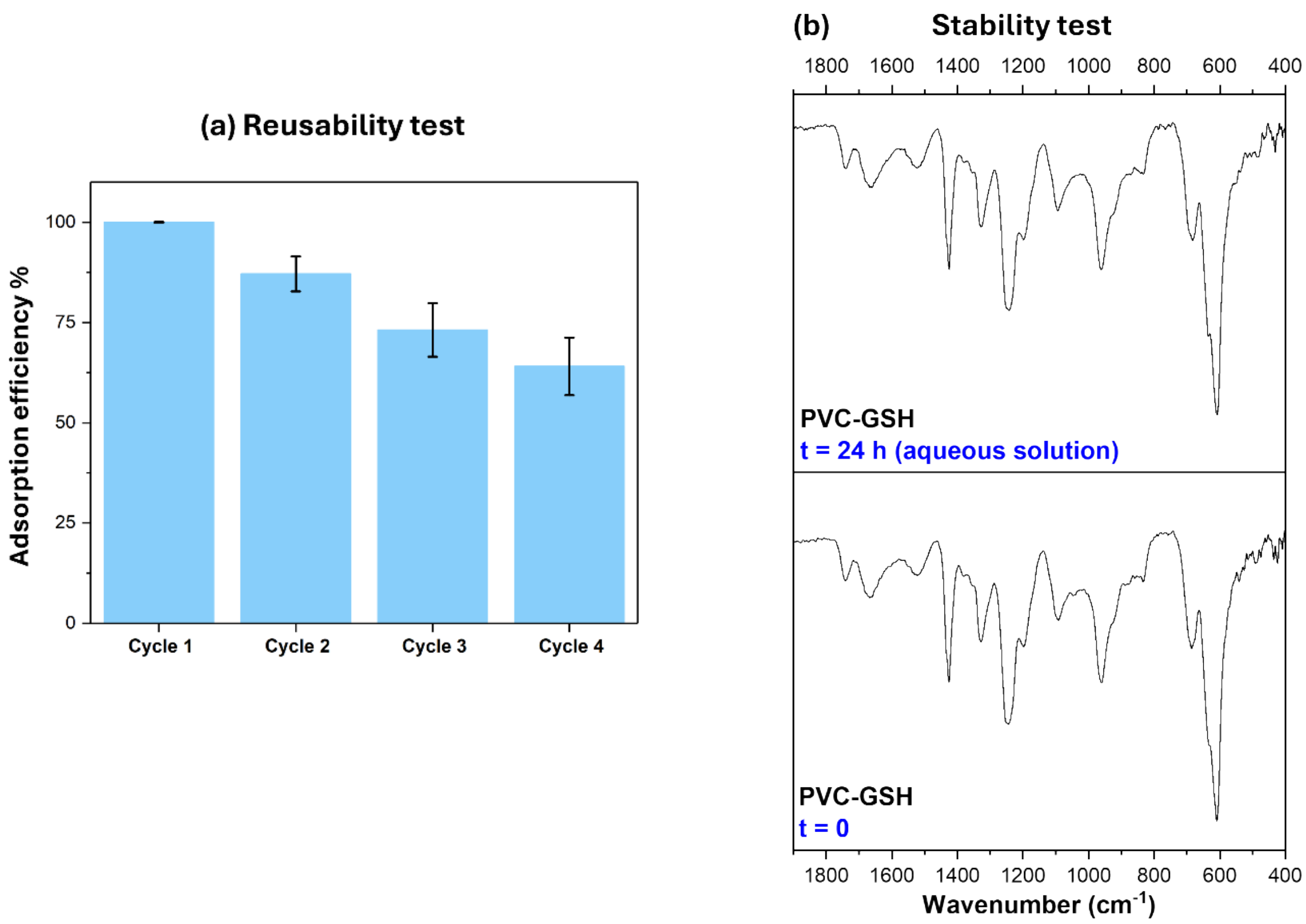

Figure S11. (a) Reusability performance of PVC-GSH over consecutive adsorption–desorption cycles (25 mg/L fluorescein, pH 6.0, 1 g/L adsorbent dose). (b) FTIR spectra of PVC-GSH before and after a 24 h stability test in 10 mL of a 10% v/v DMSO/water solution at pH 6.0 and room temperature.

X-ray photoelectron spectroscopy analysis

The average relative elemental composition of the samples is shown in Table S3. Only carbon, oxygen, nitrogen, sulfur and chlorine were detected in the samples.

Table S3. Relative concentrations of elements in PVC-GSH before and after adsorption of fluorescein as average of three positions on the sample surface.

Sample	C 1s %	Cl 2p %	N 1s %	O 1s %	S 2p %
PVC-GSH	64.91 ± 0.09	30.80 ± 0.06	1.17 ± 0.07	2.59 ± 0.21	0.54 ± 0.02
PVC-GSH + Fluorescein	66.16 ± 0.58	28.25 ± 0.62	1.18 ± 0.14	3.92 ± 0.39	0.49 ± 0.01

Table S4. C 1s component for PVC–GSH before and after adsorption of fluorescein.

	Before adsorption	After adsorption

Component (Assignment)	Binding energy	Area	Full width at half maximum	Binding energy	Area	Full width at half maximum
C–C/C–H/π-C	284.8	4145.84	1.2565	284.8	4806.06	1.0536
C–O/C–N	286.0	4495.17	1.0591	286.0	4862.68	1.3313
O–C=O	287.2	740.18	1.0999	287.8	713.03	1.2485

Table S5. O 1s component for GSH moieties and fluorescein oxygen marker in PVC-GSH before and after adsorption of fluorescein.

Component (Assignment)	Before adsorption			After adsorption		
	Binding energy	Area	Full width at half maximum	Binding energy	Area	Full width at half maximum
Carbonyl O (C=O)	531.5	513.96	1.8406	531.7	531.61	2.5749
C-O phenolic/ carboxylate (fluorescein)	532.9	1280.81	1.8461	533.1	6671.20	2.8843

Table S6. N 1s component for GSH moieties marker in PVC-GSH before and after adsorption of fluorescein.

Component (Assignment)	Before adsorption			After adsorption		
	Binding energy	Area	Full width at half maximum	Binding energy	Area	Full width at half maximum
Amide/amine	399.5	275.94	1.3676	399.5	260.23	1.1669
Protonated/ H-bonded	400.8	230.69	1.2876	400.8	317.83	1.2685

Table S7. S 2p component for GSH moieties marker in PVC-GSH before and after adsorption of fluorescein.

Component (Assignment)	Before adsorption			After adsorption		
	Binding energy	Area	Full width at half maximum	Binding energy	Area	Full width at half maximum

$2p_{3/2}$ (thiol/ thioether)	162.8	92.92	1.0175	162.8	61.08	1.0102
$2p_{1/2}$ (spin–orbit doublet)	163.8	72.29	1.0754	163.9	127.54	1.0574

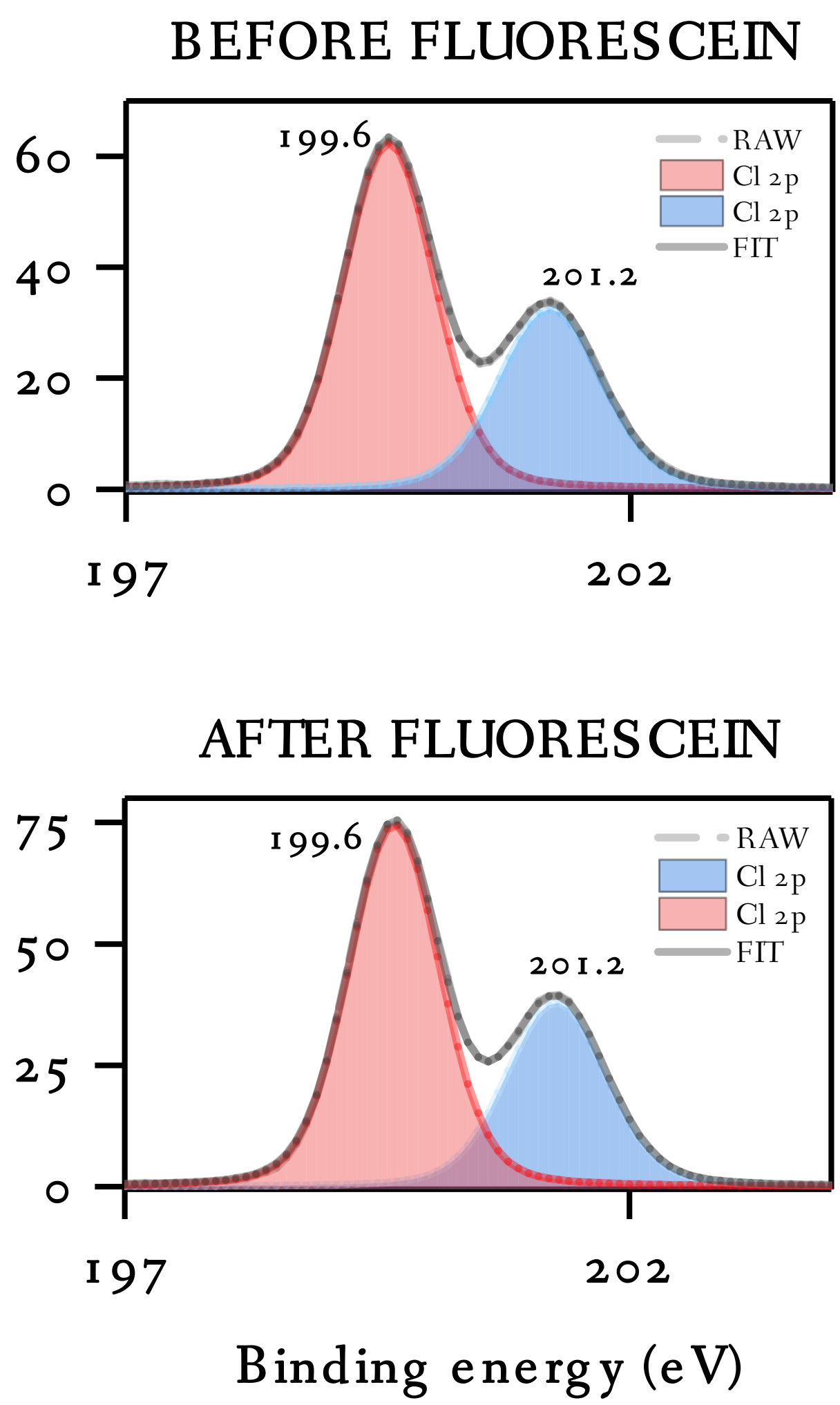

Figure S12. Binding energy of Chlorine extracted from high resolution XPS spectra in presence and absence of fluorescein.

Table S8. Cl 2p component for PVC backbone marker in PVC-GSH before and after adsorption of fluorescein.

Component (Assignment)	Before adsorption			After adsorption		
	Binding energy	Area	Full width at half maximum	Binding energy	Area	Full width at half maximum
$2p_{3/2}$	199.6	6245.88	1.0700	199.6	7130.03	1.1699

$2p_{1/2}$	201.2	4708.45	1.1079	201.2	6372.80	1.1730

Table S9. Component fractions as % of element area in PVC-GSH before and after adsorption of fluorescein.

**Element**	**Component**	**Before adsorption (%)**	**After adsorption (%)**	**Δ (fraction %)**
C 1s	C–C/C–H/π-C	53.11	46.29	−6.82
	C–O/C–N	40.26	46.84	+6.58
	O–C=O	6.63	6.87	+0.24
Cl 2p	$2p_{3/2}$	63.66	52.80	−10.85
	$2p_{1/2}$	36.34	47.20	+10.85
N 1s	Amide/amine	54.47	45.02	−9.45
	Protonated/H-bonded	45.53	54.98	+9.45
O 1s	Carbonyl (C=O)	28.64	7.38	−21.26
	C–O phenolic/ carboxylate	71.36	92.62	+21.26
S 2p	$2p_{3/2}$ (thiol/thioether)	56.25	32.38	−23.86
	$2p_{1/2}$ (spin–orbit doublet)	43.75	67.62	+23.86

Using the survey atomic percentages (Table S9) as a proxy for total peak intensity, the attenuation ratio (I/I_0) was determined to be 0.917, calculated from the reduction of chlorine from 30.80% to 28.25%.
